# GRACE-PSO: Particle Swarm Optimization with Group Rank Assessment and Cooperative Evolution

**DOI:** 10.3390/biomimetics11090664

**Published:** 2026-09-16

**Authors:** Honggang Wu, Jinxiao Li, Yufei Zhang, Lidong Gu, Pengyu Wang

**Affiliations:** 1 School of Mechanical and Electrical Engineering, Changchun University of Science and Technology, Changchun 130022, China; 2 College of Computer Science and Technology, Changchun University, Changchun 130022, China; 3 College of Instrumentation and Electrical Engineering, Jilin University, Changchun 130061, China

**Keywords:** swarm intelligence, particle swarm optimization, group rank assessment, cooperative evolution, population diversity

## Abstract

Particle swarm optimization (PSO) is a widely used bio-inspired optimization method. However, global guidance can align particle trajectories, reduce population diversity, and lead to premature convergence. To address this issue, we propose GRACE-PSO, a particle swarm optimizer based on group rank assessment and cooperative evolution. The method introduces group-best learning as an intermediate layer between personal-best and global-best learning to improve the balance between exploration and exploitation. GRACE-PSO integrates three coupled mechanisms: (1) a group-learning update that provides the population with multiple group-specific search directions; (2) a rank-based group-utility assessment that evaluates relative search effectiveness through pairwise comparisons of personal-best fitness values; and (3) a utility-driven adaptive strategy that adjusts the strengths of group-best and global-best learning and selectively reinitializes a small number of underperforming particles in stagnant groups. Experiments on 29 CEC 2017 benchmark functions at 30 and 50 dimensions against seven representative PSO methods show that GRACE-PSO achieves average ranks of 1.2414 and 1.3793, respectively. Experiments on two practical flexible intelligent metasurface optimization problems further demonstrate its competitive performance and practical applicability.

## 1. Introduction

Particle swarm optimization (PSO) is a bio-inspired method motivated by the collective behavior of bird flocks and fish schools [1,2]. Each particle represents a candidate solution and moves according to its own experience and information shared by the swarm. Owing to its gradient-free structure and few control parameters, PSO has been widely used for continuous optimization [3]. However, under the global-best topology, all particles are repeatedly guided by the same best individual. This strong social influence can gradually align particle trajectories, reduce population diversity, and lead to premature convergence on complex or multimodal problems [4]. Therefore, maintaining diverse search behaviors while preserving information sharing remains an important challenge in PSO design [5].

Existing PSO variants can be grouped into parameter control, state awareness, and redesigned learning structures. Parameter-control methods adapt inertia weights, acceleration coefficients, velocity limits, or strategy-allocation ratios during search, ranging from evolutionary-state-based adaptation to nonlinear coefficient schedules and joint control of subpopulation ratios and constriction factors [6,7]. State-aware methods infer search conditions from population distributions, evolutionary progress, or subpopulation stagnation, then modify particle behavior or trigger cooperation [8,9]. More recently, state-adaptive knowledge recall has combined dispersion-guided aggregation of elite population knowledge with stagnation-triggered reuse of historically successful search directions [10]. Redesigned learning structures alter exemplars, subpopulation organization, topology, or information-propagation paths. Cooperative PSO and comprehensive-learning PSO demonstrated the value of decomposed search and multiple historical exemplars [11,12], while recent variants introduce heterogeneous learning and multi-subswarm cooperation [9,13]. These studies enrich information sources but do not determine their evolving influence on the population.

Multi-swarm and heterogeneous designs are closely related to GRACE-PSO because they preserve distinct search roles and exchange information across subpopulations. However, the quality and convergence state of each group may change during evolution, altering the usefulness of its information. Existing schemes typically rely on sub-swarm-best improvement, stagnation, spatial relations, individual states, or level-based roles. Although informative, these signals do not directly provide a scale-insensitive assessment of the relative search utility reflected by the personal-best experience within each group. Comparisons based on raw fitness values may also depend on objective scale and extreme observations. Group cooperation may therefore benefit from summarizing group-level search information, comparing groups in a scale-insensitive manner, and using the assessment to adapt learning and diversity maintenance. This may improve information sharing while reducing redundant search by weak or stagnant groups.

Motivated by these observations, we propose GRACE-PSO, a particle swarm optimizer with group rank assessment and cooperative evolution. The population is divided into fixed, balanced groups, introducing group-best guidance as an intermediate social-learning layer between personal-best and global-best learning. At periodic intervals, pairwise rank comparisons of personal-best fitness values are used to construct a group-utility profile comprising utility similarity, advantage, and inferiority components. These collective signals adjust the relative strengths of group-best and global-best learning. Under stagnation, they also guide selective reinitialization of a few underperforming particles in a non-elite group. This closed loop helps retain multiple group-specific search directions while preserving useful search information. The main contributions are as follows:

- A group-learning update is introduced by incorporating group-best guidance between personal-best and global-best learning. The three-level rule broadens the learning sources and provides group-specific exemplars in addition to the global-best position.
- A group-utility assessment is developed from rank-based pairwise comparisons of personal-best fitness values. It combines utility similarity, advantage, and inferiority components into a scale-insensitive utility profile without relying on raw fitness magnitudes.
- A utility-driven adaptive strategy converts the group-utility profiles into population-level utility-similarity and advantage measures, adjusts group and global acceleration coefficients in opposite directions, and selectively reinitializes underperforming particles in a stagnant, non-elite group. This strategy preserves elite information while introducing alternative search directions under stagnation.

The remainder of this paper is organized as follows. Section 2 reviews standard PSO and related work. Section 3 presents the framework and main components of GRACE-PSO. Section 4 evaluates GRACE-PSO on the CEC 2017 benchmark suite and compares it with seven representative PSO algorithms. Section 5 presents the flexible intelligent metasurface application, and Section 6 concludes the paper and discusses future research.

## 2. Standard PSO and Representative Enhancement Strategies

This section begins by introducing the standard global-best PSO, followed by a review of representative enhancement strategies. Recent studies are categorized according to the principal mechanism targeted for modification, namely parameter control, state awareness, and learning structure.

### 2.1. Standard Particle Swarm Optimization

PSO is a population-based stochastic optimizer introduced by Kennedy and Eberhart [1]. In a *D*-dimensional search space, a swarm of *N* particles represents candidate solutions. At iteration *t*, particle *i* has a position $x_{i}^{t}\in R^{D}$ and a velocity $v_{i}^{t}\in R^{D}$. Its personal-best position $p_{i}^{t}$ is the best position it has visited up to iteration *t*, and $g^{t}$ is the best personal-best position in the swarm.

Using the inertia-weight formulation of Shi and Eberhart [2], standard global-best PSO updates each particle as

(1) $$\begin{array}{cc} v_{i}^{t+1}= & wv_{i}^{t}+c_{1}r_{1}⊙\left(p_{i}^{t}-x_{i}^{t}\right)+c_{2}r_{2}⊙\left(g^{t}-x_{i}^{t}\right), \end{array}$$

(2) $$\begin{array}{ccc} x_{i}^{t+1}= & x_{i}^{t}+v_{i}^{t+1}, & \end{array}$$

where *w* is the inertia weight, $c_{1}$ and $c_{2}$ are the cognitive and social acceleration coefficients, respectively, $r_{1}$ and $r_{2}$ are independent random vectors sampled uniformly from ${\left[0,1\right]}^{D}$, and ⊙ denotes element-wise multiplication. The inertia term maintains motion, the cognitive term returns a particle toward its own successful experience, and the social term attracts it toward the best solution shared by the swarm.

The global-best topology disseminates high-quality information rapidly and often gives fast early convergence. However, every particle receives the same social exemplar $g^{t}$. Repeated attraction toward this single position can align particle trajectories, concentrate the personal-best positions, and reduce the information available for continued exploration. Recent PSO research therefore modifies not only the numerical strength of the update terms, but also the information used to diagnose the search and the structure through which particles learn.

### 2.2. Representative Enhancement Strategies

Recent PSO enhancements can be broadly classified into parameter control, state awareness, and redesigned learning structures. Although these categories may overlap, they reflect the primary contribution of each study.

#### 2.2.1. Parameter Control

Parameter-control methods change numerical factors such as the inertia weight and acceleration coefficients during the search. Zhan et al. [6] derived an evolutionary factor from the population geometry, classified the search into exploration, exploitation, convergence, and jumping-out states, and adapted the inertia weight and acceleration coefficients accordingly; an elitist learning step was additionally activated in the convergence state. Watanabe and Xu [7] presented a unified construction of nonlinear time-varying inertia and acceleration coefficients with prescribed endpoint values, together with a crossing strategy whose cognitive–social crossing time and value can be designed in advance. Yin et al. [14] trained a deep deterministic policy-gradient controller that maps iteration progress, population diversity, and the duration without global-best improvement to group-wise values of the PSO coefficients. Zhao et al. [15] embedded a periodic parameter-fitness assessment in a fitness landscape-based hierarchical parallel search and adjusted each particle’s parameters to different extents according to the current stage and state. Von Eschwege and Engelbrecht [16] represented acceptable control-parameter ranges in a belief space, updated those ranges from selected particles, and resampled particle parameters while progressively excluding non-promising regions. Chen et al. [17] assigned a separate actor to each particle group and used a single critic to learn continuous group-wise parameter control, alongside intra-group, inter-group, and worst-particle mutation mechanisms. These studies reduce reliance on a single fixed parameter setting, although schedule-based designs still require prescribed trajectories and feedback-based controllers depend on the informativeness and transferability of their state signals.

#### 2.2.2. State Awareness

State-aware methods estimate the current search condition and use it to modify particle behavior, trigger cooperation, or maintain diversity. Li et al. [18] reused an evolutionary-state estimate based on positional distribution and fitness information to assign a high velocity limit during global search and a low limit during local search, and coupled it with boundary-handling rules. Yang et al. [8] detected a sub-swarm as stagnant when its local best failed to improve before the scheduled regrouping time, generated a vitality particle from population-wide historical information, and repelled particles that approached the globally best sub-swarm too closely. Xu et al. [19] constructed refraction-based quasi- and extended-opposition operators and used a diversity threshold to switch between local refinement and diversity restoration. Yang and Li [20] assessed each sub-swarm through its best solution, regrouped stagnant sub-swarms when their number reached a threshold, supplied a compensation sample when an individual sub-swarm stagnated earlier, and used competitive disturbance to improve solution accuracy. Li et al. [21] combined a competitive multi-swarm framework with convergence-guiding learning, an entropy-based local-diversity measure, and diversity-guiding learning. Tian et al. [22] used current swarm diversity to select high-level attraction or repulsion, then used fitness relative to the swarm average to choose lower-level updates involving regularly or slowly varying functions, mutation, or worst–best exemplar learning. These methods make the search responsive to observed conditions, but their decisions are primarily based on scalar diversity, individual fitness, or best-solution progress rather than a scale-insensitive assessment of the relative search utility across groups.

#### 2.2.3. Redesigned Learning Structures

Redesigned learning structures modify the sources, organization, or propagation paths of information used to guide particle updates. Wang et al. [23] first let each particle probe several positions by learning from fitter demonstrators and the population mean; the best probes formed a ranked temporary population from which two rank-dependent demonstrator subsets were selected for the second learning stage. Tao et al. [24] used fitness-peak clustering to form the initial sub-swarms, evolved them independently with comprehensive learning, merged them into a global swarm according to stagnation information, and alternated comprehensive and dimensional exemplar generation for selected particles. Sheng et al. [25] partitioned particles into fitness levels and varied level-wise sampling probabilities from exploration-oriented sampling early in the run to exploitation-oriented sampling later; each sampled particle learned from the top 100p% of its sub-swarm with an individually adapted *p*. Xu et al. [26] grouped components taken from existing PSO variants into a strategy pool and used an adaptive differential-evolution training engine to learn combinations from which the SLFPSO variant was assembled. Liu et al. [27] weighted superior personal-best positions as attractors and inferior ones as repellers so that the whole population contributed to each velocity estimate, and added an information-interaction operator to exchange information and inject useful stochasticity. Meng and Li [28] separated exploration and exploitation subpopulations: non-elite personal bests learned from elite personal bests through dynamic crossover in the exploration branch, while elite learning, intra-group learning, and inter-group perturbation were used in the exploitation branch. These approaches broaden and reorganize learning sources, but their decisions remain based mainly on individual fitness, ranks, stagnation, or learned component performance rather than an explicit group-level assessment of relative search utility for adaptive information sharing.

#### 2.2.4. Discussion

The three categories improve PSO from different perspectives. Parameter control adjusts the strength of particle movement, state awareness determines when the search strategy should change, and learning structures affect how information is obtained and shared. Some existing methods combine these ideas, but the use of rank relationships among personal-best fitness values to assess the relative search utility of persistent groups has received less attention. GRACE-PSO explores this direction by combining group-best learning with rank-based group-utility assessment. The assessment constructs a group-utility profile from utility similarity, advantage, and inferiority components without relying directly on raw fitness values. This information is then used to adjust group-best and global-best learning and to selectively reinitialize underperforming particles in an eligible stagnant, non-elite group.

## 3. GRACE-PSO: Particle Swarm Optimization with Group Rank Assessment and Cooperative Evolution

This section presents GRACE-PSO, which combines group-best learning with group-utility assessment to improve search coordination and diversity. Its three main components are introduced below: the group-learning update, the group-utility assessment, and the utility-driven adaptive strategy.

### 3.1. Overall Framework

Let *N* denote the population size. At iteration *t*, the position and velocity of particle *i* are denoted by $x_{i}^{t}$ and $v_{i}^{t}$, respectively, while its personal-best position and the global-best position are denoted by $p_{i}^{t}$ and $g^{t}$. After initialization, the population is randomly partitioned into *K* balanced groups, denoted by $G_{1},\ldots ,G_{K}$. Let $n_{k}=\left|G_{k}\right|$ be the size of group $G_{k}$, where $\sum_{k=1}^{K}n_{k}=N$. The group-best position $q_{k}^{t}$ is defined as the personal-best position with the lowest fitness in $G_{k}$.

Group membership remains fixed throughout each run, allowing different groups to maintain distinct search trajectories. At each iteration, particles update their states using personal-best, group-best, and global-best information. Every $T_{c}$ iterations, GRACE-PSO performs pairwise rank comparisons of personal-best fitness values across groups and constructs group-utility profiles comprising utility similarity, advantage, and inferiority scores. The utility-similarity and advantage scores are used to adjust the group and global acceleration coefficients, whereas the utility-similarity and inferiority scores are used to identify stagnant non-elite groups for selective reinitialization. This three-level learning framework supports population-wide information sharing while retaining group-specific search directions.

**Figure 1:**
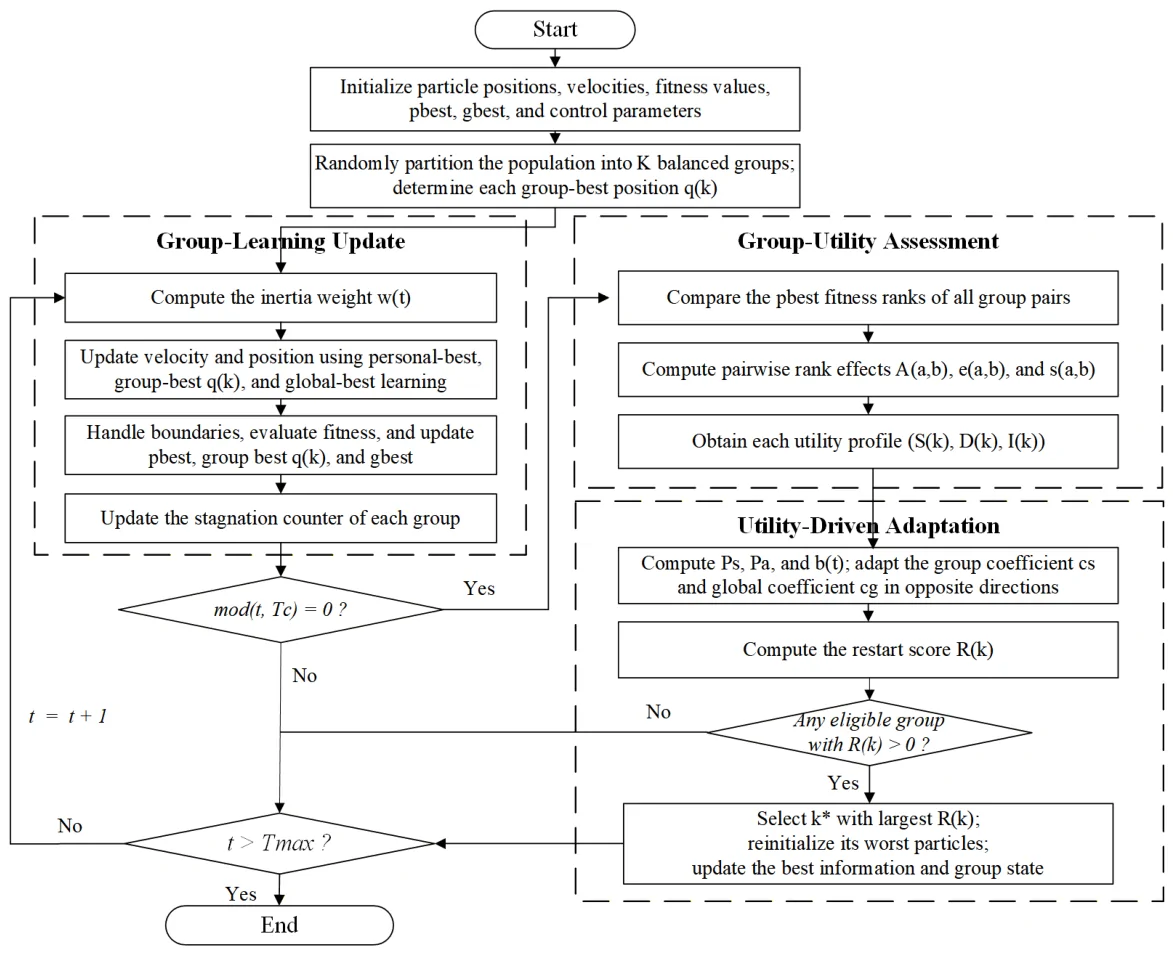
Overall flowchart of GRACE-PSO.

The overall flowchart of GRACE-PSO is shown in Figure 1.

### 3.2. Group-Learning Update

For particle *i* belonging to group $G_{k}$, the velocity is updated as

(3) $$\begin{array}{cc} v_{i}^{t+1}= & w^{t}v_{i}^{t}+c_{p}r_{1}⊙\left(p_{i}^{t}-x_{i}^{t}\right)+c_{s}^{t}r_{2}⊙\left(q_{k}^{t}-x_{i}^{t}\right)+c_{g}^{t}r_{3}⊙\left(g^{t}-x_{i}^{t}\right), \end{array}$$

where $r_{1}$, $r_{2}$, and $r_{3}$ are independent random vectors uniformly sampled from ${\left[0,1\right]}^{D}$. The coefficients $c_{p}=1.55$, $c_{s}^{t}$, and $c_{g}^{t}$ are the acceleration coefficients associated with personal-best, group-best, and global-best learning, respectively.

The inertia weight decreases linearly over the search process:

(4) $$w^{t}=w_{\max}-\frac{t-1}{T_{\max}-1}\left(w_{\max}-w_{\min}\right),$$

where $T_{\max}$ is the maximum number of iterations. The inertia limits are $w_{\max}=0.90$ and $w_{\min}=0.40$. The particle position is then updated by

(5) $$x_{i}^{t+1}=x_{i}^{t}+v_{i}^{t+1}.$$

The three learning terms have complementary roles. Personal-best learning retains the search experience of each particle, group-best learning shares a high-quality direction within its group, and global-best learning disseminates the best information across the population. Because the groups generally maintain different group-best positions, this update provides multiple intermediate search directions and reduces excessive dependence on a single global-best position.

### 3.3. Group-Utility Assessment

Raw fitness magnitudes can vary substantially across search stages. GRACE-PSO therefore evaluates each group through rank-based pairwise comparisons of personal-best fitness values. For groups $G_{a}$ and $G_{b}$, the probability of superiority of group $G_{a}$ over group $G_{b}$ is defined as

(6) $$A_{ab}=\frac{1}{n_{a}n_{b}}\sum_{i\in G_{a}}\sum_{j\in G_{b}}\left[I\left(f_{i}<f_{j}\right)+\frac{1}{2}I\left(f_{i}=f_{j}\right)\right],$$

where $f_{i}=f\left(p_{i}\right)$, and $n_{a}$ and $n_{b}$ are the sizes of the two groups. In the implementation, $A_{ab}$ is computed from the Mann–Whitney *U* statistic using average ranks for tied values.

The signed superiority effect and the corresponding pairwise utility-similarity component are defined as

(7) $$e_{ab}=2A_{ab}-1,\;s_{ab}=1-\left|e_{ab}\right|.$$

A positive $e_{ab}$ indicates that group $G_{a}$ is generally superior to group $G_{b}$. A value of $s_{ab}$ close to one indicates that neither group consistently dominates the pairwise rank comparisons and is therefore interpreted as similar rank-based search utility. This measure does not imply equality of the complete distributions, which may differ in dispersion, shape, or modality.

The group-level utility similarity, advantage, and inferiority scores of group $G_{k}$ are given by

(8a) $$\begin{array}{cc} S_{k}\; & =\frac{1}{K-1}\sum_{j\neq k}s_{kj}, \end{array}$$

(8b) $$\begin{array}{cc} D_{k}\; & =\frac{1}{K-1}\sum_{j\neq k}\max\left(0,e_{kj}\right), \end{array}$$

(8c) $$\begin{array}{cc} I_{k}\; & =\max\left(0,-\frac{1}{K-1}\sum_{j\neq k}e_{kj}\right). \end{array}$$

Here, $S_{k}$ aggregates the pairwise utility similarity between group $G_{k}$ and the other groups. $D_{k}$ reflects its relative advantage, while $I_{k}$ reflects its relative disadvantage. Together, $S_{k}$, $D_{k}$, and $I_{k}$ form the rank-based group-utility profile used by the adaptive strategy. $D_{k}$ focuses on positive pairwise effects, whereas $I_{k}$ captures negative effects. Since these measures are based on ranks rather than raw fitness values, they are not affected by the objective scale. For simplicity, all groups are assumed to meet the minimum-size requirement. Otherwise, only valid group comparisons are used in the calculation.

### 3.4. Utility-Driven Adaptive Strategy

The group-utility profiles guide two adaptive strategies: population-level coefficient adaptation and group-level selective reinitialization. For coefficient adaptation, the population-level utility-similarity and advantage measures are defined as

(9) $$P_{s}=\frac{1}{K}\sum_{k=1}^{K}S_{k},\;P_{a}=\max_{k}D_{k}.$$

Their relative strength is summarized by the adaptation balance factor

(10) $$b^{t}=\frac{P_{a}-P_{s}}{P_{a}+P_{s}+\lambda },$$

where λ>0 is a stabilizing constant and $b^{t}$ is restricted to [−1,1]. For the CEC experiments, λ=0.40. The group and global acceleration coefficients are adjusted in opposite directions:

(11) $$c_{s}^{t}=c_{s}^{0}-\alpha_{s}b^{t},\;c_{g}^{t}=c_{g}^{0}+\alpha_{g}b^{t},$$

with both coefficients restricted to predefined ranges. The group coefficient uses $c_{s}^{0}=0.95$, $\alpha_{s}=0.25$, and $c_{s}^{t}\in \left[0.75,1.20\right]$. The global coefficient uses $c_{g}^{0}=1.05$, $\alpha_{g}=0.50$, and $c_{g}^{t}\in \left[0.70,1.55\right]$. When advantage is stronger, global-best learning is reinforced to exploit high-quality information. When utility similarity is stronger, group-best learning is reinforced to promote distinct search directions among groups. The inferiority score $I_{k}$ is used only for selective reinitialization and does not enter the coefficient update.

For each stagnant non-elite group, the reinitialization score is defined as

(12) $$R_{k}=\frac{1}{2}\left[\max\left(0,S_{k}-\bar{S}\right)+\max\left(0,I_{k}-\bar{I}\right)\right],$$

where $\bar{S}$ and $\bar{I}$ are the mean utility-similarity and inferiority scores across all valid groups. A larger $R_{k}$ indicates a stronger combination of above-average utility similarity and inferiority. Among eligible stagnant groups that do not contain the current global-best particle, the group with the largest $R_{k}$ is selected and denoted by $k^{*}$; a small number of its worst-performing particles are reinitialized.

For each selected particle, several candidate positions are sampled uniformly from the search space, and the candidate farthest from the current global-best position in the normalized decision space is retained. This selective reinitialization introduces alternative search directions while protecting the group containing the global-best particle and preserving other high-quality search information.

### 3.5. Pseudocode of GRACE-PSO

Algorithm 1 summarizes the complete GRACE-PSO procedure. Here, *N* denotes the population size, $T_{c}$ is the group-utility assessment interval, and ρ is the fraction of particles reinitialized in the selected group. For the CEC experiments, ρ=0.08.

Positions are initialized uniformly in the search domain, and velocities are initialized to zero. Each velocity component is limited by $v_{\max}=0.18\left(u-l\right)$. An out-of-range position is clipped to the nearest bound, and the corresponding velocity is reversed and multiplied by 0.50. A group is considered stagnant when its group-best fitness does not improve by more than $10^{-12}\max(1,|F_{k}^{\mathrm{old}}\left|\right)$ in one iteration, where $F_{k}=f\left(q_{k}\right)$. Group-utility assessment and restart eligibility are checked every $T_{c}=\max\left(5,\mathrm{round}\left(0.02T_{\max}\right)\right)$ iterations, which gives $T_{c}=30$ and 50 for the 30- and 50-dimensional CEC experiments, respectively. Restart eligibility requires at least five particles, excludes the global-best group, and uses a 40-iteration group-specific cooldown. Three uniformly sampled candidates are considered for each selected particle, and the candidate farthest from the global best in the normalized search space is retained. Only the retained candidate is evaluated, and its velocity is sampled from $\left[-0.20v_{\max},0.20v_{\max}\right]$. All restart evaluations count toward the same hard evaluation budget.
**Algorithm 1** GRACE-PSO**Require:** Objective function *f*, population size *N*, maximum number of iterations $T_{\max}$, number of groups *K*, assessment interval $T_{c}$, and reinitialization ratio ρ
**Ensure:** Global-best solution g
1:Initialize particle positions $x_{i}$, velocities $v_{i}$, and fitness values2:Set $p_{i}\leftarrow x_{i}$ and determine the global-best position g3:Randomly partition the population into *K* balanced groups4:Determine the group-best position $q_{k}$ of each group5:Initialize $c_{s}^{t}$ and $c_{g}^{t}$ and the stagnation counter of each group6:**for** t=1$T_{\max}$**do**7:   Compute $w^{t}$ using Equation (4)8:   **for** i=1*N* **do**9:      Update $v_{i}$ using Equation (3)10:     Update $x_{i}$ using Equation (5)11:     Apply the boundary-handling rule and evaluate $f(x_{i})$12:     Update $p_{i}$ if the current position is improved13:   **end for**14:   Update the group-best positions $q_{k}$ and the global-best position g15:   Update the stagnation counter of each group16:   **if** $\mathrm{mod}(t,T_{c})=0$ **then**17:     Compute the pairwise rank effects using Equation (6)18:     Compute $S_{k}$, $D_{k}$, and $I_{k}$ using Equations (8a)–()19:     Update $c_{s}^{t}$ and $c_{g}^{t}$ using Equations (9)–(11)20:     Compute the reinitialization score $R_{k}$ using Equation (12)21:     **if** at least one eligible stagnant group exists **then**22:        Select the group $k^{*}$ with the largest $R_{k}$23:        Select the worst $\left\lceil \rho n_{k^{*}}\right\rceil$ particles in $G_{k^{*}}$24:        Reinitialize the selected particles using distant random candidates25:        Evaluate the reinitialized particles and update $p_{i}$, $q_{k}$, and g26:     **end if**27:   **end if**28:**end for**29:**return** g


### 3.6. Computational Complexity

Let *N* be the population size, *D* the problem dimension, and $C_{f}$ the cost of one fitness evaluation. In each iteration, updating all particle velocities and positions requires O(ND) operations, while evaluating the population requires $O(NC_{f})$.

The group-utility assessment performs rank comparisons among groups. A direct implementation that processes each group pair has complexity

(13) $$O\left(KN\log\frac{N}{K}\right).$$

If every group is sorted once and the ordered lists are reused, the assessment requires $O\;\left(N\log(N/K)+KN\right)$ operations.

Because group-utility assessment and selective reinitialization are performed only periodically and *K* is generally small, the dominant computational cost remains particle updating and fitness evaluation. The leading time complexity of GRACE-PSO can therefore be approximated as

(14) $$O\left[T_{\max}N\left(D+C_{f}\right)\right].$$

The particle positions, velocities, and personal-best positions each require O(ND) storage. Hence, the dominant space complexity is O(ND), which is of the same order as that of standard PSO.

## 4. Experiments on the Benchmark Test Suite

In this section, the optimization performance of GRACE-PSO is evaluated using the CEC 2017 benchmark test suite. The experiments are designed to address the following four research questions: (1) How does GRACE-PSO perform overall on different categories of test functions compared with representative PSO variants? (2) Are the performance differences among the compared algorithms statistically significant? (3) How does each algorithmic mechanism contribute to the performance of GRACE-PSO? (4) Is the performance of GRACE-PSO sensitive to its key parameter settings?

### 4.1. Experimental Setup

#### 4.1.1. Benchmark Functions

The CEC 2017 benchmark suite is widely used for evaluating algorithms for single-objective real-parameter optimization [29]. Following the official recommendations and commonly adopted experimental settings, the frequently unstable function F2 was excluded. Consequently, the performance of the algorithms was evaluated on the remaining 29 test functions, namely F1 and F3–F30. Specifically, F1 and F3 are unimodal functions, F4–F10 are simple multimodal functions, F11–F20 are hybrid functions, and F21–F30 are composition functions.

Experiments were conducted at D=30 and D=50, with the search domain ${\left[-100,100\right]}^{D}$. The optimization performance of each algorithm was measured using the best error value obtained at the end of each independent run, defined as

(15) $$\varepsilon_{f,r}=f\left(x_{f,r}^{\mathrm{best}}\right)-f_{f}^{★},\;f_{f}^{★}=100f,$$

where *f* denotes the index of the test function, *r* denotes the index of the independent run, $x_{f,r}^{\mathrm{best}}$ denotes the best solution obtained for function *f* in the *r*th run, and $f_{f}^{★}$ denotes the known global optimum fitness value of function *f*. A smaller value of $\varepsilon_{f,r}$ indicates better optimization performance. When $\varepsilon_{f,r}=0$, the algorithm has successfully reached the known global optimum of the corresponding test function.

#### 4.1.2. Comparison Algorithms

To validate the effectiveness of GRACE-PSO, seven representative particle swarm optimization algorithms were selected for comparison:

- AWPSO: An adaptive weighting strategy based on the Sigmoid function is employed to dynamically adjust the acceleration terms of particles [30]. The distance-dependent coefficients satisfy $c_{p},c_{g}\in \left[1.75,2.00\right]$, and *w* decreases from 0.90 to 0.40.
- AFMPSO: An adaptive forgetting mechanism is combined with a centroid-guided learning strategy to enhance the search capability of the swarm [31]. The implementation uses $w_{t}=2/\left(t+1\right)$, $c_{1}=c_{2}=2$, and a history length of 25.
- VPPSO: A velocity-pause mechanism, an improved velocity update term, and a dual-swarm structure are introduced to balance exploration and exploitation [32]. The settings are α=0.30, $c_{1}=c_{2}=2$, and $w_{t}=\exp\left[-{\left(2.5t/T\right)}^{2.5}\right]$.
- APSO: The inertia weight and acceleration coefficients are adaptively adjusted by identifying the evolutionary state of the swarm [6]. The fuzzy evolutionary-state estimator uses w∈[0.4,0.9] and $c_{1},c_{2}\in \left[1.5,2.5\right]$, with elitist learning in the convergence state.
- CLPSO: Dimension-wise learning exemplars are constructed using the historical best positions of different particles, thereby improving swarm diversity [12]. The implementation uses w:0.90→0.40, c=1.49445, and a refresh gap of 7.
- PSO: The classical global-best particle swarm optimization strategy with a linearly decreasing inertia weight is adopted [1,2]. The settings are w:0.90→0.40 and $c_{1}=c_{2}=2$.
- CPSO: The hybrid cooperative PSO decomposes the decision vector into five-variable components and periodically exchanges information with a full-dimensional swarm [11]. CPSO-H6 is used at D=30 and CPSO-H10 at D=50, with w:1.0→0, $c_{1}=c_{2}=1.49$, and $v_{\max}=0.5\left(u-l\right)$.

The selected comparison algorithms cover several representative directions for improving PSO, including adaptive parameter adjustment, forgetting mechanisms, velocity control, cooperative decomposition, comprehensive learning, and classical global-best learning. The comparison is limited to the implemented methods listed above.

To ensure a fair comparison, all algorithms were implemented in accordance with the algorithmic procedures described in their original publications. Their algorithm-specific settings are given above, and the common experimental settings and key GRACE-PSO parameters are summarized in Table 1.

#### 4.1.3. Evaluation Protocol

The common dimension, search-domain, population, run, seed, and evaluation-budget settings, together with the key GRACE-PSO parameters, are listed in Table 1. Initialization and additional restart evaluations were included in the hard budgets. Function-wise ranks were calculated from unrounded mean errors, with average ranks assigned only to exact ties.

All experiments were conducted in MATLAB R2024a on a 64-bit Windows 11 operating system. The hardware platform was equipped with an Intel Core i7-14700 processor and 32 GB of memory.

### 4.2. Results on the CEC 2017 Benchmark Suite

#### 4.2.1. Overall Performance

Table 2, Table 3, Table 4 and Table 5 report the function-wise mean error, standard deviation, and rank of the eight algorithms at D=30 and D=50, respectively. Table 6 summarizes the aggregate rank results at both dimensions.

At D=30, GRACE-PSO ranks first on 23 functions, second on five, and third on one. At D=50, it ranks first on 21 functions, second on six, third on one, and fourth on one. The corresponding average ranks are 1.2414 and 1.3793. The results show a similar overall rank pattern across the two dimensions, while the function-wise comparisons also indicate that GRACE-PSO is not uniformly best on every problem.

#### 4.2.2. Performance by Landscape Category at 30 Dimensions

Table 7 groups the D=30 functions by landscape type. GRACE-PSO achieves the best average rank in all four categories: 1.000 on unimodal functions, 1.143 on multimodal functions, 1.600 on hybrid functions, and 1.000 on composition functions. It ranks first on every composition function from F21 to F30. Its weaker category is the hybrid group, primarily because of F14, F15, F18, and F19, but its category-level average rank remains lower than that of every competitor.

**Figure 2:**
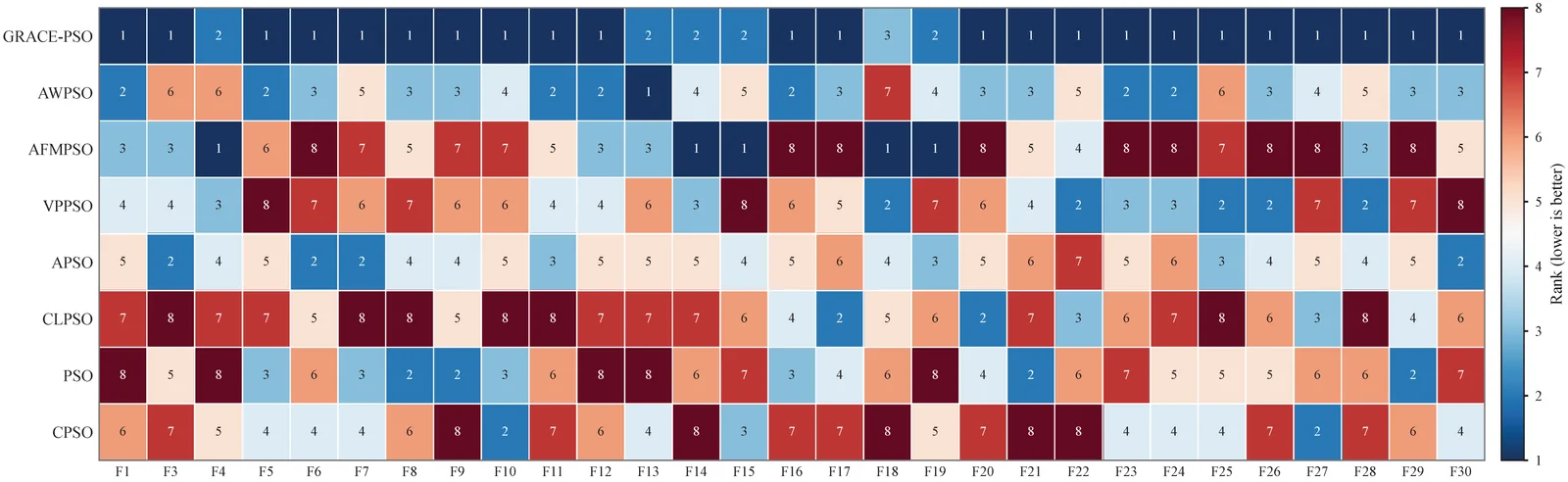
Function-by-algorithm ranks on the 29 CEC 2017 functions at D=30. Ranks are calculated from the unrounded mean errors; blue denotes lower ranks, red denotes higher ranks, and exact ties receive average ranks.

Figure 2 shows the function-wise ranks at D=30.

#### 4.2.3. Overall Statistical Comparison

Table 8 reports the omnibus tests for the eight algorithms. Significant differences are present in both dimensions. Table 9 gives two-sided Wilcoxon signed-rank comparisons across the 29 unrounded function means, with Holm correction over the seven comparisons against GRACE-PSO. The function-level columns summarize two-sided rank-sum tests on the 30-run samples, with Holm correction over the 29 functions within each competitor.

#### 4.2.4. Convergence and Distribution Analysis

**Figure 3:**
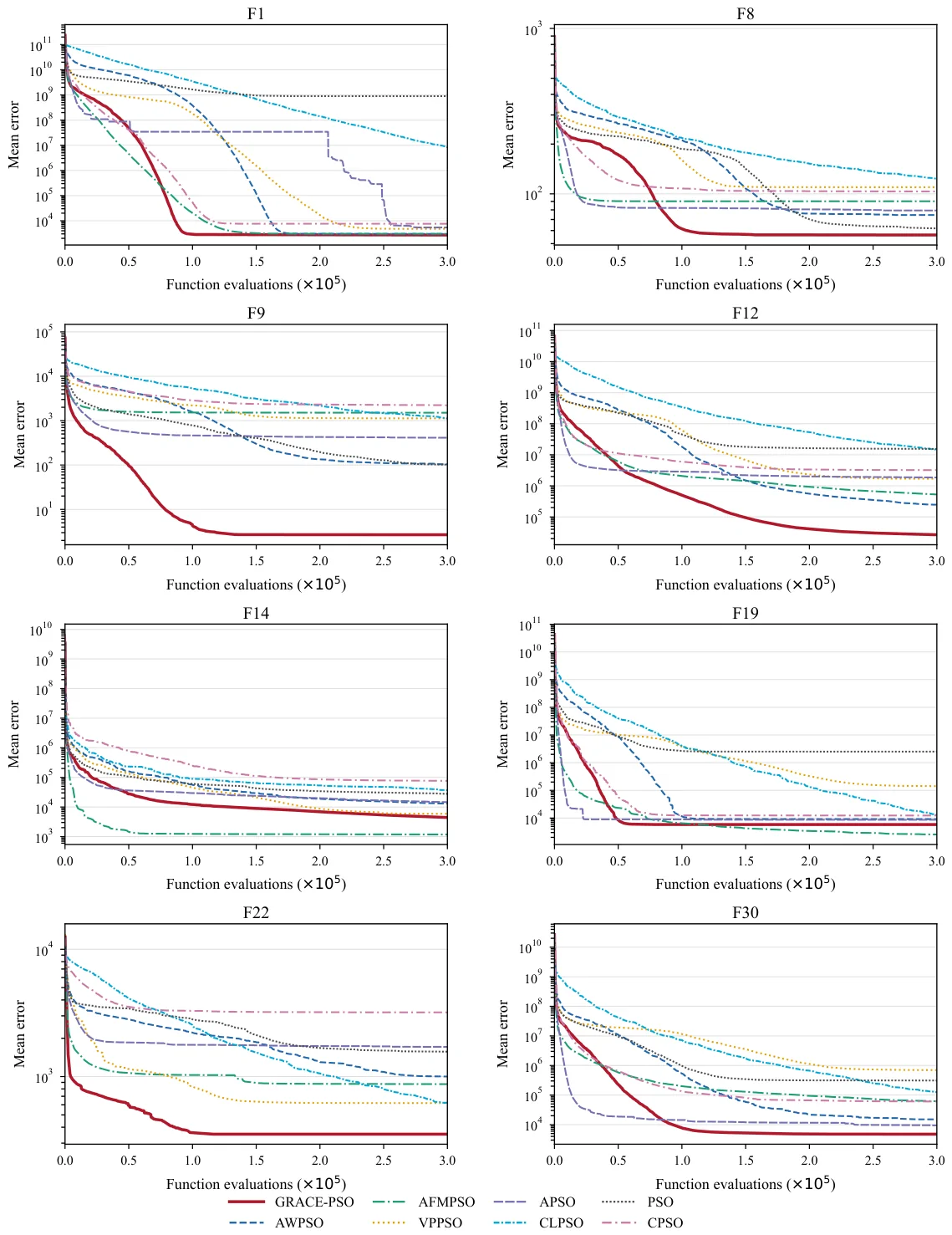
Mean convergence curves over 30 independent runs on eight representative CEC 2017 functions at D=30.

**Figure 4:**
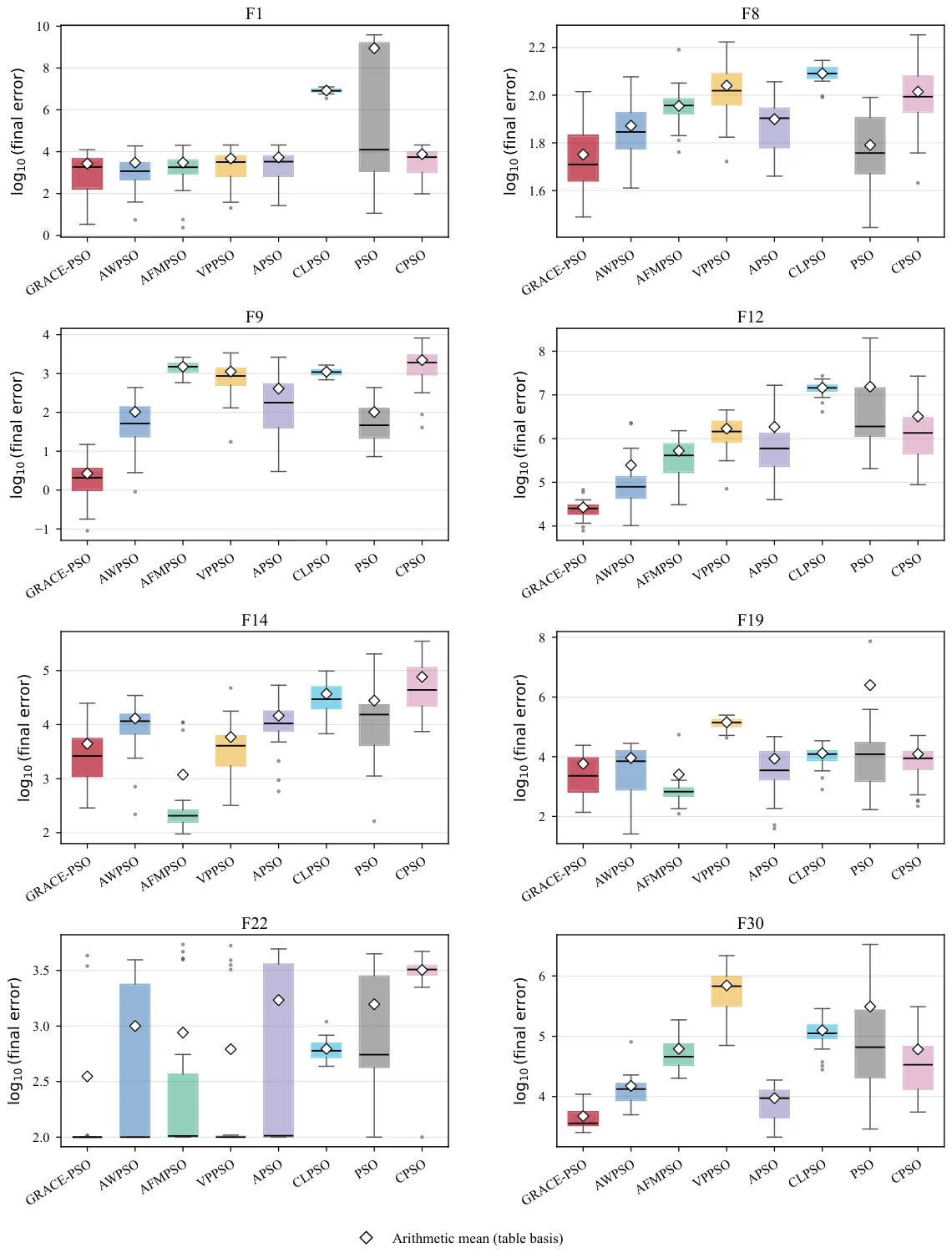
Distributions of the final errors on a $\log_{10}$ scale for eight representative CEC 2017 functions at D=30. Diamonds denote the arithmetic means used in Table 2 and Table 3, whereas gray circles denote observations beyond 1.5 times the interquartile range.

At D=30, Figure 3 and Figure 4 compare all eight algorithms on eight representative functions, including unimodal, multimodal, hybrid, and composition problems. The selected functions cover both the strong performance and representative exceptions of GRACE-PSO.

The convergence curves are plotted against the number of objective function evaluations to ensure a fair comparison among algorithms with different evaluation costs per iteration. GRACE-PSO achieves the lowest final mean error on six of the eight functions. On F12 and F14, it maintains clear improvement in the later search stage, showing its ability to continue exploiting promising regions. On F8, F19, and F22, the curves stabilize earlier. For F14 and F19, AFMPSO obtains lower final errors, which is consistent with the numerical results. Overall, the convergence results confirm the strong performance of GRACE-PSO across different landscapes while also showing its limitations on specific functions.

The box plots in Figure 4 show the final errors on a $\log_{10}$ scale, which allows results spanning several orders of magnitude to be compared clearly. Lower medians indicate better performance, while narrower boxes indicate smaller variability. The diamonds denote the logarithm of the arithmetic mean computed on the original error scale. For display only, zero errors are replaced by $10^{-12}$ before the logarithm is taken; the unmodified errors are used in all tables and tests.

GRACE-PSO exhibits favorable medians and distributions on F8, F9, F12, F22, and F30. F1 illustrates the difference between mean- and median-based summaries: GRACE-PSO has the lowest arithmetic mean, whereas AWPSO has the lowest median. A similar distinction is observed on F22, where several algorithms frequently reach an error level near $10^{2}$ but also produce long upper tails. On F14 and F19, AFMPSO performs best according to both the mean and the median. These observations demonstrate the value of reporting both mean-based rankings and distributional results.

### 4.3. Intergroup Structure Diagnostic

**Figure 5:**
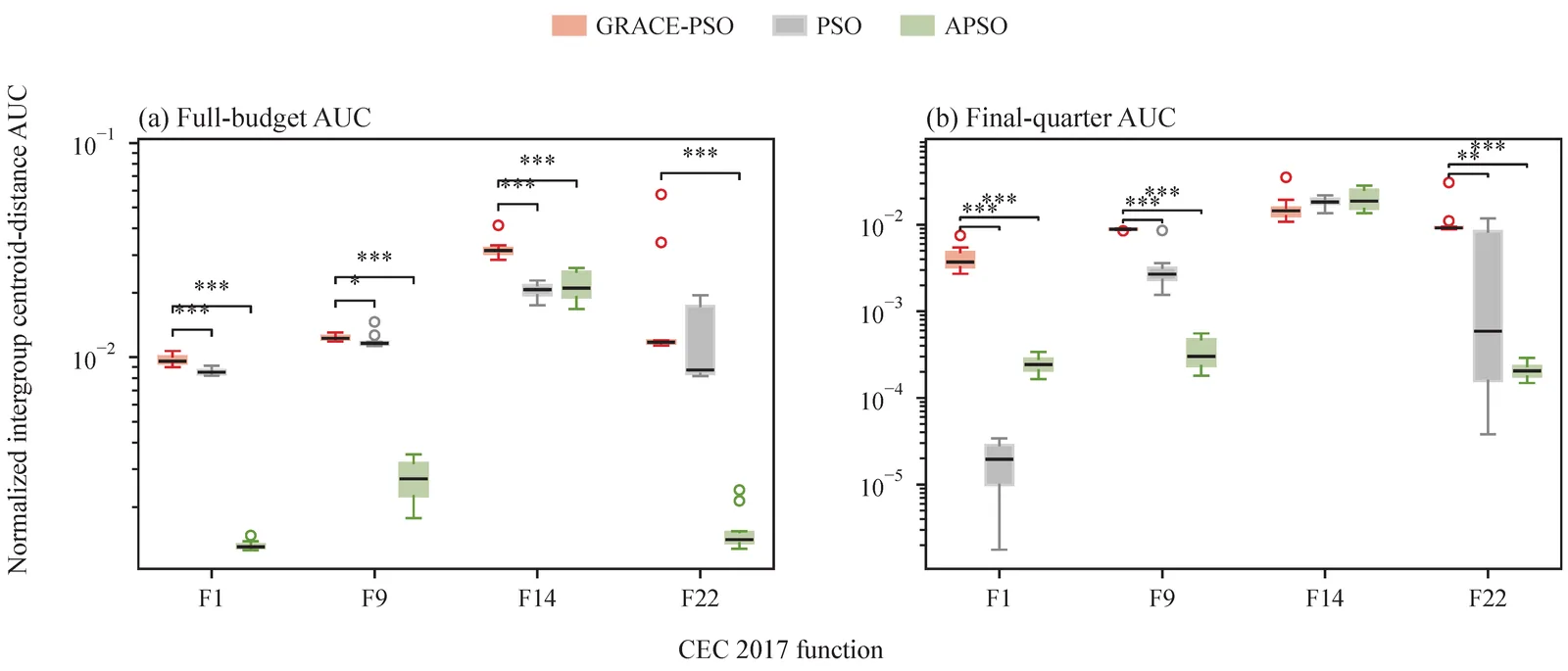
Run-level distributions of intergroup centroid-distance AUC for GRACE-PSO, PSO, and APSO at D=30: (**a**) the full evaluation budget and (**b**) the final 25% of the budget. Results are based on ten runs, and the vertical axis is logarithmic. Circles denote observations beyond 1.5 times the interquartile range. Brackets denote comparisons with GRACE-PSO; *, **, and indicate Holm-adjusted p<0.05, p<0.01, and p<0.001, respectively.

To examine whether GRACE-PSO maintains distinct group-level search structures during evolution, we use the intergroup centroid distance. At each evaluation point, particle coordinates are normalized by their search ranges, and the mean pairwise Euclidean distance between the six group centroids is computed and divided by $\sqrt{D}$. Table 10 reports the area under the resulting distance curve (AUC) over the full evaluation budget and its final 25% on F1, F9, F14, and F22. Figure 5 shows the corresponding run-level distributions. For PSO and APSO, the same fixed partition is used for this diagnostic calculation.

Over the full evaluation budget, GRACE-PSO has a higher intergroup centroid-distance AUC than both controls on all four functions; the differences are significant after Holm correction in three comparisons with PSO and all four comparisons with APSO. During the final 25% of the budget, GRACE-PSO retains higher AUC on F1, F9, and F22, whereas F14 shows lower AUC than both controls without a significant difference ($p_{H}=0.0757$). At restart-assessment points, the median improvement in centroid-distance change relative to non-restart assessment points is 0.03261, 0.03228, 0.01749, and 0.03365 on F1, F9, F14, and F22, respectively ($p_{H}\leq 0.01562$). These results indicate that GRACE-PSO generally maintains more persistent intergroup separation on the tested functions, while not implying uniformly higher whole-population diversity.

### 4.4. Ablation Study

To examine the roles of the main components of GRACE-PSO, six configurations are compared: standard PSO; fixed grouping with group-best learning only (GroupOnly); group learning with coefficient adaptation (GL+CA); group learning with selective reinitialization (GL+SR); complete GRACE-PSO; and the complete framework using group-best improvement and stagnation duration instead of the rank-based group-utility assessment (NonRank). All configurations are evaluated over 30 runs on the 29 functions at D=30 under the same 300,000-evaluation budget. The results are reported in Table 11.

GRACE-PSO achieves the best aggregate rank among the six configurations and outperforms each reduced variant on 18–20 of the 29 functions. The improvement over standard PSO is significant after Holm correction, confirming the overall benefit of the proposed group-based framework. The differences among the structurally related GRACE-PSO variants are smaller and do not reach the corrected 0.05 threshold, indicating that no single adaptive component alone accounts for the overall performance pattern. The rank-based configuration also shows a lower average rank and 20/9 wins/losses against NonRank, providing a descriptive advantage for the proposed group-utility assessment.

### 4.5. Parameter Sensitivity Analysis

A one-factor-at-a-time analysis is conducted for the three main parameters of GRACE-PSO. The reference setting is K=6, ρ=0.08, and λ=0.40. Each parameter is varied over five values while the other two remain fixed: K∈{2,4,6,8,10}, ρ∈{0.04,0.06,0.08,0.10,0.12}, and λ∈{0.20,0.30,0.40,0.60,0.80}. Each configuration uses 30 runs with seeds 1001–1030 at D=30 and 300,000 evaluations, matching the main comparison. The reference-setting columns use the same 30 run records as the main experiment. The reference setting was fixed during preliminary development on CEC 2017, so this analysis is interpreted as a post hoc local robustness check rather than independent parameter selection.

#### 4.5.1. Sensitivity to the Number of Groups

The parameter *K* controls the number of groups and, consequently, the amount of information represented by each group-utility profile. Increasing *K* creates more but smaller groups, which can diversify group-best guidance but also makes each rank-based group-utility estimate depend on fewer particles. Table 12 is designed to reveal whether this tradeoff is consistent across the benchmark suite or concentrated in particular landscape categories.

Within the *K* family, the average ranks are 4.4138, 2.9655, 2.6897, 2.3448, and 2.5862 for K=2,4,6,8,10, respectively. The smallest grouping level is the least reliable setting, while K=8 has the best aggregate rank and 12 first places. The reference value K=6 obtains 22 top-three finishes. The category-level results are not monotonic: K=6 has the lowest average rank on the unimodal subset, K=8 on the multimodal and hybrid subsets, and K=10 on the composition subset.

#### 4.5.2. Sensitivity to the Reinitialization Ratio

The parameter ρ determines the fraction of poorly performing particles reinitialized in the selected stagnant group. A small value produces a mild diversity injection, whereas a large value increases exploration but may discard useful local search information. Table 13 therefore compares five ratios spanning 0.04–0.12 to determine whether the reference setting balances stagnation recovery and local-search continuity.

For the reinitialization ratio, the average ranks are 3.1724, 3.1379, 3.1379, 2.7931, and 2.7586 for ρ=0.04,0.06,0.08,0.10,0.12, respectively. The results do not show a monotonic response. The reference ρ=0.08 is tied with ρ=0.06 in average rank, while ρ=0.12 and ρ=0.10 rank first and second overall.

#### 4.5.3. Sensitivity to the Adaptation Damping Parameter

The parameter λ acts as a damping constant in the denominator of the balance factor in Equation (10). Reducing λ increases the magnitude of the balance factor and therefore produces a stronger redistribution of group-best and global-best learning pressure. Conversely, increasing λ attenuates the response to group-utility differences and makes coefficient adaptation more conservative. Table 14 is intended to determine whether a moderate response provides a better exploration–exploitation balance than either a weak or an aggressive response.

For the adaptation damping parameter, the average ranks are 2.7586, 3.3103, 3.0345, 2.9310, and 2.9655 for λ=0.20,0.30,0.40,0.60,0.80, respectively. The best value varies across function categories: λ=0.60 ranks first on the unimodal subset, the reference value on the multimodal subset, λ=0.30 on the hybrid subset, and λ=0.20 on the composition subset. Since the analysis varies one parameter at a time on a single benchmark suite, these results are used only to assess parameter robustness.

## 5. Application to Flexible Intelligent Metasurface Design

Flexible intelligent metasurfaces (FIMs) extend conventional reconfigurable intelligent surfaces by allowing both the element positions and reflection phases to be adjusted [33]. This additional spatial degree of freedom can improve channel shaping, but it also couples element movement with phase control. As a result, the received power depends jointly on element positions and reflection phases, while the element positions must also satisfy physical spacing constraints. The resulting design problem is therefore nonlinear, nonconvex, and constrained.

### 5.1. Problem Formulation

To evaluate GRACE-PSO in this setting, we consider two joint position–phase optimization problems, denoted FIM-1 and FIM-2. FIM-1 serves as the basic four-element case, while FIM-2 increases the number of elements, propagation paths, decision variables, and spacing constraints, resulting in a more challenging optimization problem.

Let *N* denote the number of metasurface elements, and let $p_{n}={\left[x_{n},z_{n}\right]}^{T}$ and $v_{n}$ denote the position and reflection phase of element *n*, respectively. For *L* base-station paths and *P* receiver paths, the equivalent channel is

(16) $$\begin{array}{cc} h(y)= & \frac{1}{\sqrt{LP}}\sum_{n=1}^{N}e^{jv_{n}}\sum_{l=1}^{L}\sum_{p=1}^{P}\alpha_{l}\beta_{p}^{*}\\ \; & \times \exp\;\left\{j\frac{2\pi }{\lambda }\left[\left(\theta_{B,l}-\theta_{R,p}\right)z_{n}+\left(\phi_{B,l}-\phi_{R,p}\right)x_{n}\right]\right\}+\gamma , \end{array}$$

where $\alpha_{l}$, $\beta_{p}$, and γ are complex channel coefficients, $\theta_{B,l}$, $\phi_{B,l}$, $\theta_{R,p}$, and $\phi_{R,p}$ are direction parameters, and λ is the wavelength. The received power is

(17) $$P_{\mathrm{rx}}\left(y\right)={\left|h\left(y\right)\right|}^{2},\;y={\left[x_{1},\ldots ,x_{N},z_{1},\ldots ,z_{N},v_{1},\ldots ,v_{N}\right]}^{T}.$$

The variables satisfy

(18) $$x_{n},z_{n}\in \left[-2\lambda ,2\lambda \right],\;v_{n}\in \left[0,2\pi \right],\;{∥p_{i}-p_{j}∥}_{2}\geq d_{\min}\;\left(i<j\right),$$

where $d_{\min}=\lambda /2$. The carrier frequency is 10 GHz, giving λ=0.03 m.

To make results comparable across channel realizations, the received power is normalized by

(19) $$P_{\mathrm{UB}}={\left(\frac{N}{\sqrt{LP}}\sum_{l=1}^{L}\sum_{p=1}^{P}\left|\alpha_{l}\beta_{p}^{*}\right|+\left|\gamma \right|\right)}^{2},\;\eta =\min\;\left(1,\frac{P_{\mathrm{rx}}}{P_{\mathrm{UB}}}\right).$$

Let CV be the sum of the normalized bound and inter-element spacing violations. Every optimizer uses the same feasibility-first fitness:

(20) $$F\left(y\right)=\left\{\begin{array}{cc} 1-\eta , & \mathrm{CV}\leq 10^{-12},\\ 1+\mathrm{CV}, & \mathrm{CV}>10^{-12}. \end{array}\right.$$

Thus, every feasible solution is preferred to every infeasible solution, feasible solutions are ordered by received power, and infeasible solutions are ordered by constraint violation without a tunable penalty coefficient. For statistical analysis, infeasible solutions are assigned $\tilde{\eta }=0$, while feasible solutions use $\tilde{\eta }=\eta$.

### 5.2. Experimental Protocol

Table 15 summarizes the settings of the two FIM problems. The channel coefficients are independently drawn from CN(0,1), while the direction parameters are drawn from U[−1,1]. Thirty channel realizations are generated and shared by all algorithms to ensure a fair comparison. FIM-1 contains four elements with L=P=3, whereas FIM-2 increases the problem size to eight elements with L=P=6.

GRACE-PSO is compared with AWPSO, AFMPSO, VPPSO, APSO, CLPSO, CPSO, and standard PSO. All algorithms use a population size of 100 and a maximum budget of 10,000 objective-function evaluations per run. The evaluation count includes initialization and any additional candidate evaluations, ensuring the same effective budget for all methods. Each algorithm is independently run 20 times on each of the 30 channel realizations for both problems. GRACE-PSO uses K=6, ρ=0.08, λ=0.40, the coefficient settings in Section 3.2 and Section 3.4, $T_{c}=5$, and the same boundary and restart rules described in Section 3.5.

The fixed-position phase-beamforming baseline places the elements on a centered rectangular grid with adjacent spacing $d_{\min}$ and sets each phase in closed form to align its reflected term with the direct channel. It uses the same 30 channel realizations and does not optimize element positions.

For each algorithm and channel realization, the median result over the 20 runs is used for statistical comparison. The resulting 30 paired observations are analyzed using the Friedman test and average ranks. Pairwise comparisons between GRACE-PSO and each competitor are further performed using the two-sided Wilcoxon signed-rank test, together with the rank-biserial correlation and the numbers of wins, ties, and losses. The reported gain in decibels is obtained by computing $10\log_{10}\left(\tilde{\eta }/\eta_{\mathrm{PBF}}\right)$ for each run, taking the median over the 20 runs for each channel, and then taking the median over the 30 channel realizations.

### 5.3. Results and Discussion

Table 16 summarizes the results on the two FIM problems. GRACE-PSO obtains median adjusted normalized powers of 0.7054 and 0.1769 on FIM-1 and FIM-2, respectively. Its average ranks are 2.900 and 2.433, placing it among the leading methods together with APSO, PSO, and CPSO. Its median gains over the fixed-position phase-beamforming baseline reach 6.248 dB and 7.902 dB on FIM-1 and FIM-2, respectively.

The Friedman tests confirm significant overall differences among the algorithms on both FIM-1 ($p=9.21\times 10^{-31}$) and FIM-2 ($p=3.00\times 10^{-37}$). As shown in Table 17, GRACE-PSO significantly outperforms AFMPSO, VPPSO, and CLPSO on FIM-1. On FIM-2, it significantly outperforms AWPSO, AFMPSO, VPPSO, CLPSO, and PSO. Its differences from APSO and CPSO are not significant on either problem. In particular, GRACE-PSO outperforms PSO on 21 of the 30 FIM-2 channel realizations, with a Holm-adjusted p=0.0055 and a rank-biserial correlation of 0.652.

**Figure 6:**
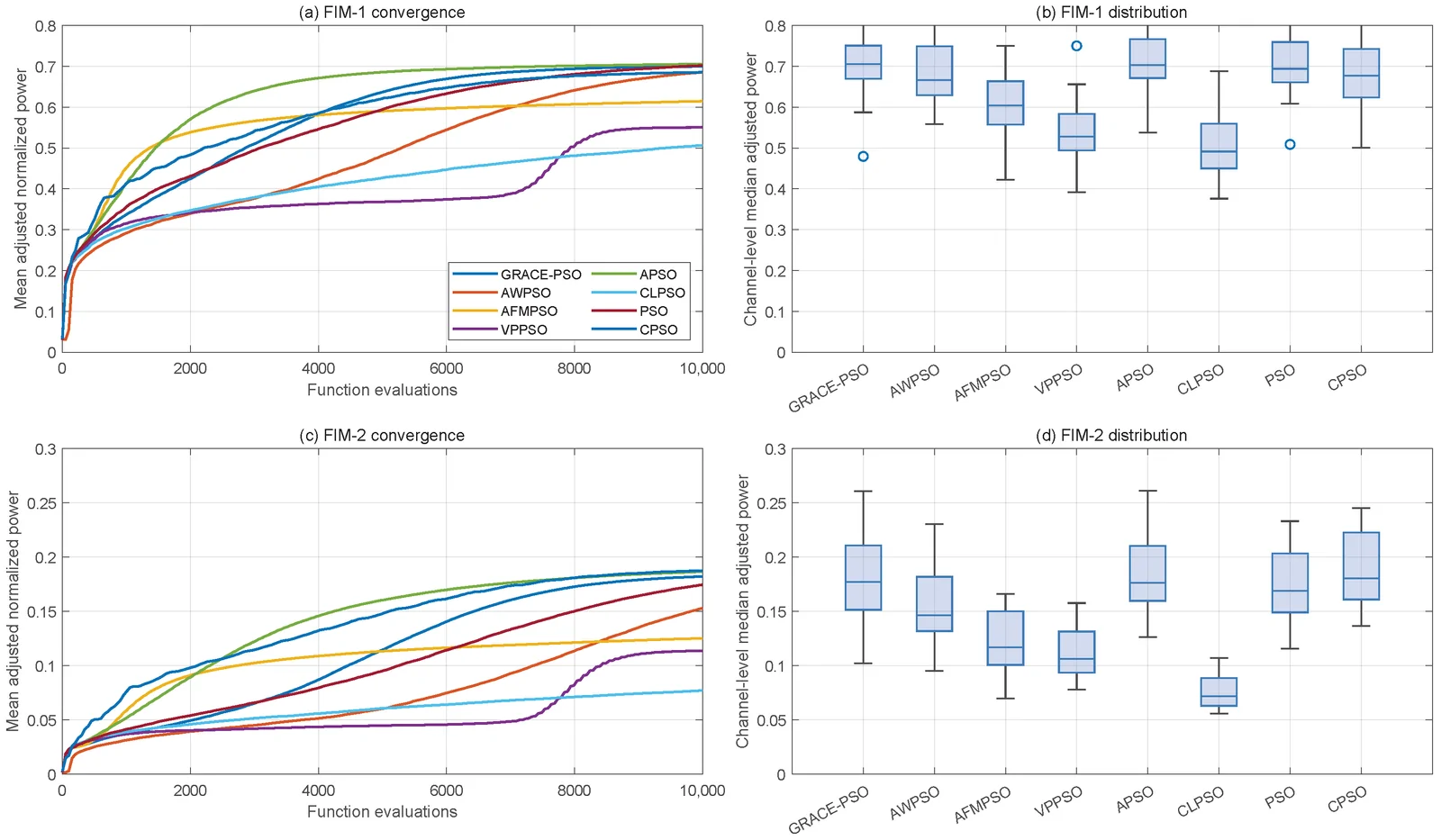
Convergence curves and channel-level distributions on FIM-1 and FIM-2. Curves show the mean adjusted normalized power over all independent runs, and box plots show the 30 channel-level medians.

Across the two problems, GRACE-PSO achieves the second-best overall average rank of 2.667, behind APSO at 2.283 and slightly ahead of CPSO at 2.767. Figure 6 further illustrates the convergence behavior and channel-level distributions. The results support competitive solution quality and reliable feasibility on the two tested FIM cases. The CEC 2017 suite contains diverse unconstrained test functions and uses a substantially larger evaluation budget, whereas the two FIM cases share the same structured wireless optimization model, include active spacing constraints, and use a budget of 10,000 evaluations. Under these conditions, several algorithms reach similar high-quality feasible regions, which narrows the between-method differences.

## 6. Conclusions and Future Work

This paper proposed GRACE-PSO, integrating group-based learning, rank-based group-utility assessment, and utility-driven adaptation. By combining personal-best, group-best, and global-best information with adaptive coefficients and selective reinitialization, GRACE-PSO supports group-specific search structures while retaining global guidance. Mechanism diagnostics show more persistent intergroup separation than PSO and APSO on most tested functions. On 29 CEC 2017 functions, GRACE-PSO achieved average ranks of 1.2414 at D=30 and 1.3793 at D=50, with the lowest mean error on 23 and 21 functions, respectively. Corrected pairwise tests showed significant differences from all seven comparison algorithms at both dimensions. On the FIM problems, GRACE-PSO produced feasible solutions and remained statistically competitive with the leading methods, supporting its practical applicability to constrained joint position–phase optimization.

Future work will focus on three directions. First, GRACE-PSO will be evaluated on higher-dimensional, dynamic, and multi-objective optimization problems. Second, adaptive group sizes and utility-assessment intervals will be explored to further improve search flexibility. Third, the FIM application will be extended to measured channel models and hardware-based validation.

## Figures and Tables

**Table 1:** Common settings and key GRACE-PSO parameters used in the CEC 2017 experiments.

Setting	Value
Dimension	D=30 and D=50
Search domain	${\left[-100,100\right]}^{D}$
Population size	200 particles
Independent runs	30, with seeds 1001–1030
Evaluation budget	300,000 evaluations at D=30 and 500,000 at D=50
Key GRACE-PSO parameters	K=6, ρ=0.08, and λ=0.40
Assessment interval	$T_{c}=30$ at D=30 and $T_{c}=50$ at D=50

**Table 2:** Experimental results on the 30-dimensional CEC 2017 benchmark functions (Part 1 of 2).

Function		GRACE-PSO	AWPSO	AFMPSO	VPPSO	APSO	CLPSO	PSO	CPSO
F1	Mean	$2.715\times 10^{3}$	$2.997\times 10^{3}$	$3.030\times 10^{3}$	$4.754\times 10^{3}$	$5.407\times 10^{3}$	$8.412\times 10^{6}$	$8.924\times 10^{8}$	$7.590\times 10^{3}$
	Std	$2.935\times 10^{3}$	$4.334\times 10^{3}$	$3.905\times 10^{3}$	$5.590\times 10^{3}$	$6.490\times 10^{3}$	$1.867\times 10^{6}$	$1.188\times 10^{9}$	$7.464\times 10^{3}$
	Rank	1	2	3	4	5	7	8	6
F3	Mean	$1.800\times 10^{-3}$	$3.312\times 10^{3}$	$1.132\times 10^{2}$	$1.214\times 10^{2}$	$1.131\times 10^{1}$	$8.107\times 10^{4}$	$1.826\times 10^{3}$	$2.738\times 10^{4}$
	Std	$8.385\times 10^{-3}$	$1.092\times 10^{3}$	$6.198\times 10^{2}$	$6.650\times 10^{2}$	$1.004\times 10^{1}$	$1.269\times 10^{4}$	$5.399\times 10^{2}$	$1.471\times 10^{4}$
	Rank	1	6	3	4	2	8	5	7
F4	Mean	$7.012\times 10^{1}$	$1.353\times 10^{2}$	$6.883\times 10^{1}$	$8.172\times 10^{1}$	$8.390\times 10^{1}$	$1.518\times 10^{2}$	$1.648\times 10^{2}$	$1.082\times 10^{2}$
	Std	$3.012\times 10^{1}$	$3.179\times 10^{1}$	$3.486\times 10^{1}$	$2.237\times 10^{1}$	$2.088\times 10^{1}$	$1.153\times 10^{1}$	$7.627\times 10^{1}$	$2.763\times 10^{1}$
	Rank	2	6	1	3	4	7	8	5
F5	Mean	$4.991\times 10^{1}$	$5.984\times 10^{1}$	$1.142\times 10^{2}$	$1.200\times 10^{2}$	$8.810\times 10^{1}$	$1.185\times 10^{2}$	$6.388\times 10^{1}$	$8.786\times 10^{1}$
	Std	$1.481\times 10^{1}$	$1.445\times 10^{1}$	$2.354\times 10^{1}$	$3.478\times 10^{1}$	$1.918\times 10^{1}$	$8.850\times 10^{0}$	$1.324\times 10^{1}$	$1.802\times 10^{1}$
	Rank	1	2	6	8	5	7	3	4
F6	Mean	$2.353\times 10^{-2}$	$8.026\times 10^{-2}$	$3.505\times 10^{1}$	$1.958\times 10^{1}$	$2.862\times 10^{-2}$	$1.260\times 10^{0}$	$1.291\times 10^{0}$	$7.404\times 10^{-1}$
	Std	$5.461\times 10^{-2}$	$1.508\times 10^{-1}$	$6.763\times 10^{0}$	$8.177\times 10^{0}$	$5.044\times 10^{-2}$	$1.651\times 10^{-1}$	$1.463\times 10^{0}$	$9.152\times 10^{-1}$
	Rank	1	3	8	7	2	5	6	4
F7	Mean	$8.700\times 10^{1}$	$1.237\times 10^{2}$	$1.544\times 10^{2}$	$1.375\times 10^{2}$	$1.050\times 10^{2}$	$1.574\times 10^{2}$	$1.054\times 10^{2}$	$1.128\times 10^{2}$
	Std	$2.057\times 10^{1}$	$2.223\times 10^{1}$	$3.079\times 10^{1}$	$2.886\times 10^{1}$	$1.759\times 10^{1}$	$9.018\times 10^{0}$	$2.446\times 10^{1}$	$1.798\times 10^{1}$
	Rank	1	5	7	6	2	8	3	4
F8	Mean	$5.640\times 10^{1}$	$7.449\times 10^{1}$	$9.021\times 10^{1}$	$1.097\times 10^{2}$	$7.933\times 10^{1}$	$1.234\times 10^{2}$	$6.183\times 10^{1}$	$1.034\times 10^{2}$
	Std	$1.841\times 10^{1}$	$2.061\times 10^{1}$	$1.772\times 10^{1}$	$2.867\times 10^{1}$	$2.044\times 10^{1}$	$1.013\times 10^{1}$	$1.977\times 10^{1}$	$3.218\times 10^{1}$
	Rank	1	3	5	7	4	8	2	6
F9	Mean	$2.689\times 10^{0}$	$1.035\times 10^{2}$	$1.506\times 10^{3}$	$1.126\times 10^{3}$	$4.084\times 10^{2}$	$1.111\times 10^{3}$	$1.023\times 10^{2}$	$2.216\times 10^{3}$
	Std	$2.846\times 10^{0}$	$1.184\times 10^{2}$	$5.034\times 10^{2}$	$9.050\times 10^{2}$	$5.721\times 10^{2}$	$2.690\times 10^{2}$	$1.206\times 10^{2}$	$1.788\times 10^{3}$
	Rank	1	3	7	6	4	5	2	8
F10	Mean	$2.892\times 10^{3}$	$3.004\times 10^{3}$	$3.822\times 10^{3}$	$3.313\times 10^{3}$	$3.134\times 10^{3}$	$4.382\times 10^{3}$	$2.915\times 10^{3}$	$2.910\times 10^{3}$
	Std	$5.086\times 10^{2}$	$7.974\times 10^{2}$	$7.732\times 10^{2}$	$7.660\times 10^{2}$	$5.588\times 10^{2}$	$3.053\times 10^{2}$	$5.688\times 10^{2}$	$4.650\times 10^{2}$
	Rank	1	4	7	6	5	8	3	2
F11	Mean	$7.220\times 10^{1}$	$1.061\times 10^{2}$	$1.300\times 10^{2}$	$1.235\times 10^{2}$	$1.116\times 10^{2}$	$2.540\times 10^{2}$	$1.513\times 10^{2}$	$1.664\times 10^{2}$
	Std	$2.786\times 10^{1}$	$6.085\times 10^{1}$	$3.598\times 10^{1}$	$3.414\times 10^{1}$	$4.482\times 10^{1}$	$2.621\times 10^{1}$	$5.281\times 10^{1}$	$6.067\times 10^{1}$
	Rank	1	2	5	4	3	8	6	7
F12	Mean	$2.669\times 10^{4}$	$2.466\times 10^{5}$	$5.276\times 10^{5}$	$1.694\times 10^{6}$	$1.860\times 10^{6}$	$1.460\times 10^{7}$	$1.546\times 10^{7}$	$3.211\times 10^{6}$
	Std	$1.308\times 10^{4}$	$5.559\times 10^{5}$	$4.496\times 10^{5}$	$1.050\times 10^{6}$	$3.925\times 10^{6}$	$4.932\times 10^{6}$	$3.693\times 10^{7}$	$5.419\times 10^{6}$
	Rank	1	2	3	4	5	7	8	6
F13	Mean	$1.218\times 10^{4}$	$1.116\times 10^{4}$	$1.758\times 10^{4}$	$1.164\times 10^{5}$	$2.403\times 10^{4}$	$1.167\times 10^{6}$	$3.618\times 10^{6}$	$2.291\times 10^{4}$
	Std	$1.339\times 10^{4}$	$2.639\times 10^{4}$	$1.167\times 10^{4}$	$4.946\times 10^{4}$	$2.425\times 10^{4}$	$6.852\times 10^{5}$	$1.301\times 10^{7}$	$2.260\times 10^{4}$
	Rank	2	1	3	6	5	7	8	4
F14	Mean	$4.422\times 10^{3}$	$1.302\times 10^{4}$	$1.182\times 10^{3}$	$5.891\times 10^{3}$	$1.460\times 10^{4}$	$3.712\times 10^{4}$	$2.783\times 10^{4}$	$7.641\times 10^{4}$
	Std	$5.215\times 10^{3}$	$9.524\times 10^{3}$	$3.018\times 10^{3}$	$8.748\times 10^{3}$	$1.249\times 10^{4}$	$2.356\times 10^{4}$	$4.367\times 10^{4}$	$7.779\times 10^{4}$
	Rank	2	4	1	3	5	7	6	8
F15	Mean	$4.169\times 10^{3}$	$1.467\times 10^{4}$	$3.219\times 10^{3}$	$3.868\times 10^{4}$	$1.169\times 10^{4}$	$1.516\times 10^{4}$	$2.547\times 10^{4}$	$1.103\times 10^{4}$
	Std	$4.766\times 10^{3}$	$1.242\times 10^{4}$	$3.250\times 10^{3}$	$2.981\times 10^{4}$	$1.462\times 10^{4}$	$9.175\times 10^{3}$	$2.546\times 10^{4}$	$1.166\times 10^{4}$
	Rank	2	5	1	8	4	6	7	3

**Table 3:** Experimental results on the 30-dimensional CEC 2017 benchmark functions (Part 2 of 2).

Function		GRACE-PSO	AWPSO	AFMPSO	VPPSO	APSO	CLPSO	PSO	CPSO
F16	Mean	$5.877\times 10^{2}$	$6.653\times 10^{2}$	$1.077\times 10^{3}$	$9.692\times 10^{2}$	$9.375\times 10^{2}$	$7.169\times 10^{2}$	$6.936\times 10^{2}$	$9.821\times 10^{2}$
	Std	$2.508\times 10^{2}$	$2.300\times 10^{2}$	$2.930\times 10^{2}$	$2.367\times 10^{2}$	$3.182\times 10^{2}$	$1.349\times 10^{2}$	$2.466\times 10^{2}$	$3.572\times 10^{2}$
	Rank	1	2	8	6	5	4	3	7
F17	Mean	$1.499\times 10^{2}$	$2.100\times 10^{2}$	$4.037\times 10^{2}$	$3.043\times 10^{2}$	$3.369\times 10^{2}$	$1.870\times 10^{2}$	$2.416\times 10^{2}$	$3.482\times 10^{2}$
	Std	$7.387\times 10^{1}$	$1.148\times 10^{2}$	$2.139\times 10^{2}$	$1.634\times 10^{2}$	$1.931\times 10^{2}$	$4.371\times 10^{1}$	$1.145\times 10^{2}$	$2.049\times 10^{2}$
	Rank	1	3	8	5	6	2	4	7
F18	Mean	$1.202\times 10^{5}$	$3.992\times 10^{5}$	$1.512\times 10^{4}$	$1.051\times 10^{5}$	$2.505\times 10^{5}$	$2.930\times 10^{5}$	$3.700\times 10^{5}$	$7.457\times 10^{5}$
	Std	$9.047\times 10^{4}$	$3.262\times 10^{5}$	$1.821\times 10^{4}$	$7.487\times 10^{4}$	$2.235\times 10^{5}$	$1.131\times 10^{5}$	$3.917\times 10^{5}$	$7.803\times 10^{5}$
	Rank	3	7	1	2	4	5	6	8
F19	Mean	$5.829\times 10^{3}$	$9.154\times 10^{3}$	$2.554\times 10^{3}$	$1.427\times 10^{5}$	$8.622\times 10^{3}$	$1.311\times 10^{4}$	$2.508\times 10^{6}$	$1.237\times 10^{4}$
	Std	$7.296\times 10^{3}$	$9.135\times 10^{3}$	$9.889\times 10^{3}$	$5.205\times 10^{4}$	$1.061\times 10^{4}$	$8.004\times 10^{3}$	$1.350\times 10^{7}$	$1.300\times 10^{4}$
	Rank	2	4	1	7	3	6	8	5
F20	Mean	$1.947\times 10^{2}$	$2.077\times 10^{2}$	$4.720\times 10^{2}$	$3.894\times 10^{2}$	$3.247\times 10^{2}$	$2.030\times 10^{2}$	$2.140\times 10^{2}$	$4.544\times 10^{2}$
	Std	$5.686\times 10^{1}$	$1.022\times 10^{2}$	$1.360\times 10^{2}$	$1.024\times 10^{2}$	$1.616\times 10^{2}$	$4.892\times 10^{1}$	$8.667\times 10^{1}$	$1.748\times 10^{2}$
	Rank	1	3	8	6	5	2	4	7
F21	Mean	$2.534\times 10^{2}$	$2.715\times 10^{2}$	$3.030\times 10^{2}$	$2.967\times 10^{2}$	$3.048\times 10^{2}$	$3.094\times 10^{2}$	$2.690\times 10^{2}$	$3.157\times 10^{2}$
	Std	$1.313\times 10^{1}$	$1.880\times 10^{1}$	$2.109\times 10^{1}$	$2.275\times 10^{1}$	$2.898\times 10^{1}$	$3.531\times 10^{1}$	$1.871\times 10^{1}$	$2.953\times 10^{1}$
	Rank	1	3	5	4	6	7	2	8
F22	Mean	$3.527\times 10^{2}$	$1.001\times 10^{3}$	$8.724\times 10^{2}$	$6.192\times 10^{2}$	$1.712\times 10^{3}$	$6.229\times 10^{2}$	$1.570\times 10^{3}$	$3.195\times 10^{3}$
	Std	$9.667\times 10^{2}$	$1.436\times 10^{3}$	$1.638\times 10^{3}$	$1.376\times 10^{3}$	$1.941\times 10^{3}$	$1.367\times 10^{2}$	$1.518\times 10^{3}$	$7.850\times 10^{2}$
	Rank	1	5	4	2	7	3	6	8
F23	Mean	$4.104\times 10^{2}$	$4.105\times 10^{2}$	$6.050\times 10^{2}$	$4.627\times 10^{2}$	$4.774\times 10^{2}$	$4.838\times 10^{2}$	$4.839\times 10^{2}$	$4.662\times 10^{2}$
	Std	$1.800\times 10^{1}$	$1.894\times 10^{1}$	$8.107\times 10^{1}$	$4.023\times 10^{1}$	$4.034\times 10^{1}$	$1.046\times 10^{1}$	$4.014\times 10^{1}$	$2.530\times 10^{1}$
	Rank	1	2	8	3	5	6	7	4
F24	Mean	$4.740\times 10^{2}$	$4.852\times 10^{2}$	$6.778\times 10^{2}$	$5.203\times 10^{2}$	$5.888\times 10^{2}$	$6.064\times 10^{2}$	$5.811\times 10^{2}$	$5.698\times 10^{2}$
	Std	$1.637\times 10^{1}$	$2.633\times 10^{1}$	$7.442\times 10^{1}$	$2.960\times 10^{1}$	$6.157\times 10^{1}$	$1.129\times 10^{1}$	$3.682\times 10^{1}$	$3.954\times 10^{1}$
	Rank	1	2	8	3	6	7	5	4
F25	Mean	$3.876\times 10^{2}$	$4.095\times 10^{2}$	$4.154\times 10^{2}$	$3.929\times 10^{2}$	$3.947\times 10^{2}$	$4.223\times 10^{2}$	$4.013\times 10^{2}$	$4.001\times 10^{2}$
	Std	$2.140\times 10^{0}$	$2.186\times 10^{1}$	$2.495\times 10^{1}$	$1.735\times 10^{1}$	$1.636\times 10^{1}$	$3.993\times 10^{0}$	$1.989\times 10^{1}$	$1.517\times 10^{1}$
	Rank	1	6	7	2	3	8	5	4
F26	Mean	$1.245\times 10^{3}$	$1.576\times 10^{3}$	$2.737\times 10^{3}$	$1.484\times 10^{3}$	$1.674\times 10^{3}$	$2.191\times 10^{3}$	$1.832\times 10^{3}$	$2.249\times 10^{3}$
	Std	$6.272\times 10^{2}$	$4.695\times 10^{2}$	$1.570\times 10^{3}$	$1.039\times 10^{3}$	$1.008\times 10^{3}$	$2.124\times 10^{2}$	$5.718\times 10^{2}$	$5.611\times 10^{2}$
	Rank	1	3	8	2	4	6	5	7
F27	Mean	$5.119\times 10^{2}$	$5.317\times 10^{2}$	$6.226\times 10^{2}$	$5.571\times 10^{2}$	$5.354\times 10^{2}$	$5.291\times 10^{2}$	$5.423\times 10^{2}$	$5.273\times 10^{2}$
	Std	$9.677\times 10^{0}$	$1.420\times 10^{1}$	$5.929\times 10^{1}$	$3.711\times 10^{1}$	$1.682\times 10^{1}$	$3.254\times 10^{0}$	$2.768\times 10^{1}$	$1.238\times 10^{1}$
	Rank	1	4	8	7	5	3	6	2
F28	Mean	$3.571\times 10^{2}$	$4.511\times 10^{2}$	$4.159\times 10^{2}$	$3.661\times 10^{2}$	$4.216\times 10^{2}$	$5.651\times 10^{2}$	$5.105\times 10^{2}$	$5.605\times 10^{2}$
	Std	$5.337\times 10^{1}$	$3.705\times 10^{1}$	$3.191\times 10^{1}$	$5.310\times 10^{1}$	$3.014\times 10^{1}$	$1.624\times 10^{1}$	$7.106\times 10^{1}$	$9.288\times 10^{1}$
	Rank	1	5	3	2	4	8	6	7
F29	Mean	$5.694\times 10^{2}$	$7.149\times 10^{2}$	$1.194\times 10^{3}$	$1.137\times 10^{3}$	$8.060\times 10^{2}$	$7.326\times 10^{2}$	$6.663\times 10^{2}$	$9.931\times 10^{2}$
	Std	$6.690\times 10^{1}$	$1.314\times 10^{2}$	$2.368\times 10^{2}$	$2.293\times 10^{2}$	$1.816\times 10^{2}$	$7.157\times 10^{1}$	$1.344\times 10^{2}$	$2.220\times 10^{2}$
	Rank	1	3	8	7	5	4	2	6
F30	Mean	$4.756\times 10^{3}$	$1.504\times 10^{4}$	$6.186\times 10^{4}$	$6.972\times 10^{5}$	$9.419\times 10^{3}$	$1.258\times 10^{5}$	$3.103\times 10^{5}$	$6.068\times 10^{4}$
	Std	$2.321\times 10^{3}$	$1.326\times 10^{4}$	$4.350\times 10^{4}$	$4.864\times 10^{5}$	$5.308\times 10^{3}$	$6.348\times 10^{4}$	$6.609\times 10^{5}$	$7.149\times 10^{4}$
	Rank	1	3	5	8	2	6	7	4

**Table 4:** Experimental results on the 50-dimensional CEC 2017 benchmark functions (Part 1 of 2).

Function		GRACE-PSO	AWPSO	AFMPSO	VPPSO	APSO	CLPSO	PSO	CPSO
F1	Mean	$4.022\times 10^{3}$	$1.303\times 10^{4}$	$7.453\times 10^{3}$	$4.301\times 10^{3}$	$7.414\times 10^{3}$	$1.017\times 10^{7}$	$4.102\times 10^{9}$	$6.213\times 10^{7}$
	Std	$4.062\times 10^{3}$	$9.258\times 10^{3}$	$7.816\times 10^{3}$	$6.011\times 10^{3}$	$7.356\times 10^{3}$	$2.129\times 10^{6}$	$2.814\times 10^{9}$	$2.369\times 10^{8}$
	Rank	1	5	4	2	3	6	8	7
F3	Mean	$7.159\times 10^{2}$	$3.608\times 10^{4}$	$3.438\times 10^{2}$	$6.124\times 10^{1}$	$1.439\times 10^{3}$	$1.878\times 10^{5}$	$1.318\times 10^{4}$	$7.263\times 10^{4}$
	Std	$4.462\times 10^{2}$	$1.006\times 10^{4}$	$7.781\times 10^{2}$	$3.354\times 10^{2}$	$3.076\times 10^{3}$	$1.841\times 10^{4}$	$3.926\times 10^{3}$	$2.702\times 10^{4}$
	Rank	3	6	2	1	4	8	5	7
F4	Mean	$1.148\times 10^{2}$	$2.733\times 10^{2}$	$1.293\times 10^{2}$	$1.410\times 10^{2}$	$1.122\times 10^{2}$	$2.226\times 10^{2}$	$3.254\times 10^{2}$	$1.999\times 10^{2}$
	Std	$4.833\times 10^{1}$	$7.041\times 10^{1}$	$5.918\times 10^{1}$	$5.264\times 10^{1}$	$5.015\times 10^{1}$	$1.577\times 10^{1}$	$1.181\times 10^{2}$	$9.896\times 10^{1}$
	Rank	2	7	3	4	1	6	8	5
F5	Mean	$1.112\times 10^{2}$	$1.410\times 10^{2}$	$2.269\times 10^{2}$	$2.655\times 10^{2}$	$1.911\times 10^{2}$	$2.640\times 10^{2}$	$1.529\times 10^{2}$	$2.008\times 10^{2}$
	Std	$2.453\times 10^{1}$	$2.667\times 10^{1}$	$2.819\times 10^{1}$	$4.980\times 10^{1}$	$4.537\times 10^{1}$	$1.514\times 10^{1}$	$2.903\times 10^{1}$	$3.735\times 10^{1}$
	Rank	1	2	6	8	4	7	3	5
F6	Mean	$2.250\times 10^{-1}$	$2.057\times 10^{0}$	$4.489\times 10^{1}$	$3.050\times 10^{1}$	$8.259\times 10^{-2}$	$7.840\times 10^{-1}$	$3.996\times 10^{0}$	$6.996\times 10^{-1}$
	Std	$5.197\times 10^{-1}$	$1.568\times 10^{0}$	$4.800\times 10^{0}$	$6.016\times 10^{0}$	$1.553\times 10^{-1}$	$8.292\times 10^{-2}$	$2.110\times 10^{0}$	$7.822\times 10^{-1}$
	Rank	2	5	8	7	1	4	6	3
F7	Mean	$2.012\times 10^{2}$	$3.068\times 10^{2}$	$4.310\times 10^{2}$	$2.874\times 10^{2}$	$2.311\times 10^{2}$	$3.303\times 10^{2}$	$2.320\times 10^{2}$	$2.324\times 10^{2}$
	Std	$3.143\times 10^{1}$	$4.753\times 10^{1}$	$7.080\times 10^{1}$	$7.157\times 10^{1}$	$4.333\times 10^{1}$	$1.255\times 10^{1}$	$4.161\times 10^{1}$	$4.196\times 10^{1}$
	Rank	1	6	8	5	2	7	3	4
F8	Mean	$1.080\times 10^{2}$	$1.360\times 10^{2}$	$2.275\times 10^{2}$	$2.446\times 10^{2}$	$1.851\times 10^{2}$	$2.689\times 10^{2}$	$1.366\times 10^{2}$	$1.971\times 10^{2}$
	Std	$2.345\times 10^{1}$	$2.965\times 10^{1}$	$3.208\times 10^{1}$	$6.042\times 10^{1}$	$4.433\times 10^{1}$	$1.804\times 10^{1}$	$2.940\times 10^{1}$	$4.209\times 10^{1}$
	Rank	1	2	6	7	4	8	3	5
F9	Mean	$7.140\times 10^{1}$	$6.670\times 10^{2}$	$6.369\times 10^{3}$	$4.592\times 10^{3}$	$3.978\times 10^{3}$	$4.743\times 10^{3}$	$1.618\times 10^{3}$	$1.292\times 10^{4}$
	Std	$1.348\times 10^{2}$	$3.695\times 10^{2}$	$1.301\times 10^{3}$	$2.857\times 10^{3}$	$2.339\times 10^{3}$	$6.809\times 10^{2}$	$9.201\times 10^{2}$	$5.241\times 10^{3}$
	Rank	1	2	7	5	4	6	3	8
F10	Mean	$5.259\times 10^{3}$	$6.244\times 10^{3}$	$6.765\times 10^{3}$	$6.210\times 10^{3}$	$5.527\times 10^{3}$	$8.835\times 10^{3}$	$5.414\times 10^{3}$	$5.225\times 10^{3}$
	Std	$1.126\times 10^{3}$	$1.158\times 10^{3}$	$8.353\times 10^{2}$	$8.774\times 10^{2}$	$8.613\times 10^{2}$	$3.445\times 10^{2}$	$8.395\times 10^{2}$	$7.199\times 10^{2}$
	Rank	2	6	7	5	4	8	3	1
F11	Mean	$1.590\times 10^{2}$	$2.687\times 10^{2}$	$2.641\times 10^{2}$	$1.884\times 10^{2}$	$2.009\times 10^{2}$	$4.593\times 10^{2}$	$3.189\times 10^{2}$	$3.231\times 10^{2}$
	Std	$4.660\times 10^{1}$	$7.177\times 10^{1}$	$7.559\times 10^{1}$	$3.514\times 10^{1}$	$4.857\times 10^{1}$	$5.326\times 10^{1}$	$9.256\times 10^{1}$	$1.387\times 10^{2}$
	Rank	1	5	4	2	3	8	6	7
F12	Mean	$4.581\times 10^{5}$	$4.130\times 10^{6}$	$6.810\times 10^{6}$	$3.405\times 10^{6}$	$2.569\times 10^{7}$	$7.745\times 10^{7}$	$8.941\times 10^{8}$	$1.281\times 10^{7}$
	Std	$3.257\times 10^{5}$	$4.658\times 10^{6}$	$1.336\times 10^{7}$	$2.409\times 10^{6}$	$4.948\times 10^{7}$	$1.470\times 10^{7}$	$1.231\times 10^{9}$	$2.033\times 10^{7}$
	Rank	1	3	4	2	6	7	8	5
F13	Mean	$2.733\times 10^{3}$	$8.605\times 10^{3}$	$2.815\times 10^{4}$	$9.437\times 10^{4}$	$9.367\times 10^{5}$	$3.300\times 10^{6}$	$8.729\times 10^{7}$	$2.116\times 10^{4}$
	Std	$3.325\times 10^{3}$	$8.569\times 10^{3}$	$1.382\times 10^{4}$	$7.025\times 10^{4}$	$2.822\times 10^{6}$	$1.397\times 10^{6}$	$1.958\times 10^{8}$	$1.801\times 10^{4}$
	Rank	1	2	4	5	6	7	8	3
F14	Mean	$2.415\times 10^{4}$	$1.038\times 10^{5}$	$4.177\times 10^{4}$	$2.932\times 10^{4}$	$1.051\times 10^{5}$	$5.698\times 10^{5}$	$2.031\times 10^{5}$	$7.652\times 10^{5}$
	Std	$2.209\times 10^{4}$	$6.781\times 10^{4}$	$1.222\times 10^{5}$	$2.283\times 10^{4}$	$1.363\times 10^{5}$	$2.547\times 10^{5}$	$2.809\times 10^{5}$	$7.657\times 10^{5}$
	Rank	1	4	3	2	5	7	6	8
F15	Mean	$5.030\times 10^{3}$	$6.175\times 10^{3}$	$7.654\times 10^{3}$	$3.454\times 10^{4}$	$7.889\times 10^{3}$	$5.271\times 10^{4}$	$4.323\times 10^{4}$	$1.185\times 10^{4}$
	Std	$5.103\times 10^{3}$	$6.038\times 10^{3}$	$5.921\times 10^{3}$	$1.946\times 10^{4}$	$9.318\times 10^{3}$	$2.248\times 10^{4}$	$4.113\times 10^{4}$	$7.047\times 10^{3}$
	Rank	1	2	3	6	4	8	7	5

**Table 5:** Experimental results on the 50-dimensional CEC 2017 benchmark functions (Part 2 of 2).

Function		GRACE-PSO	AWPSO	AFMPSO	VPPSO	APSO	CLPSO	PSO	CPSO
F16	Mean	$9.661\times 10^{2}$	$1.232\times 10^{3}$	$1.804\times 10^{3}$	$1.394\times 10^{3}$	$1.593\times 10^{3}$	$1.329\times 10^{3}$	$1.299\times 10^{3}$	$1.835\times 10^{3}$
	Std	$3.347\times 10^{2}$	$3.185\times 10^{2}$	$3.434\times 10^{2}$	$4.763\times 10^{2}$	$3.067\times 10^{2}$	$1.743\times 10^{2}$	$4.279\times 10^{2}$	$3.814\times 10^{2}$
	Rank	1	2	7	5	6	4	3	8
F17	Mean	$9.885\times 10^{2}$	$1.048\times 10^{3}$	$1.421\times 10^{3}$	$1.383\times 10^{3}$	$1.279\times 10^{3}$	$9.173\times 10^{2}$	$1.183\times 10^{3}$	$1.527\times 10^{3}$
	Std	$2.671\times 10^{2}$	$2.863\times 10^{2}$	$3.102\times 10^{2}$	$3.279\times 10^{2}$	$2.758\times 10^{2}$	$1.191\times 10^{2}$	$2.514\times 10^{2}$	$3.501\times 10^{2}$
	Rank	2	3	7	6	5	1	4	8
F18	Mean	$1.011\times 10^{5}$	$1.687\times 10^{6}$	$1.157\times 10^{5}$	$1.387\times 10^{5}$	$1.197\times 10^{6}$	$1.897\times 10^{6}$	$2.028\times 10^{6}$	$2.710\times 10^{6}$
	Std	$4.497\times 10^{4}$	$2.034\times 10^{6}$	$1.774\times 10^{5}$	$6.774\times 10^{4}$	$2.291\times 10^{6}$	$8.123\times 10^{5}$	$1.972\times 10^{6}$	$2.671\times 10^{6}$
	Rank	1	5	2	3	4	6	7	8
F19	Mean	$1.391\times 10^{4}$	$1.348\times 10^{4}$	$1.215\times 10^{4}$	$1.315\times 10^{5}$	$1.553\times 10^{4}$	$1.356\times 10^{4}$	$5.317\times 10^{5}$	$1.473\times 10^{4}$
	Std	$9.130\times 10^{3}$	$1.396\times 10^{4}$	$1.383\times 10^{4}$	$6.313\times 10^{4}$	$1.144\times 10^{4}$	$5.769\times 10^{3}$	$1.176\times 10^{6}$	$1.914\times 10^{4}$
	Rank	4	2	1	7	6	3	8	5
F20	Mean	$6.131\times 10^{2}$	$7.434\times 10^{2}$	$1.089\times 10^{3}$	$9.871\times 10^{2}$	$9.010\times 10^{2}$	$6.237\times 10^{2}$	$6.394\times 10^{2}$	$1.316\times 10^{3}$
	Std	$2.422\times 10^{2}$	$2.731\times 10^{2}$	$2.785\times 10^{2}$	$2.487\times 10^{2}$	$2.854\times 10^{2}$	$1.297\times 10^{2}$	$2.499\times 10^{2}$	$3.062\times 10^{2}$
	Rank	1	4	7	6	5	2	3	8
F21	Mean	$3.074\times 10^{2}$	$3.296\times 10^{2}$	$4.442\times 10^{2}$	$4.466\times 10^{2}$	$3.812\times 10^{2}$	$4.791\times 10^{2}$	$3.646\times 10^{2}$	$4.258\times 10^{2}$
	Std	$2.108\times 10^{1}$	$2.467\times 10^{1}$	$3.715\times 10^{1}$	$5.705\times 10^{1}$	$3.275\times 10^{1}$	$1.826\times 10^{1}$	$3.851\times 10^{1}$	$4.458\times 10^{1}$
	Rank	1	2	6	7	4	8	3	5
F22	Mean	$5.499\times 10^{3}$	$6.398\times 10^{3}$	$7.837\times 10^{3}$	$5.774\times 10^{3}$	$6.449\times 10^{3}$	$7.754\times 10^{3}$	$5.782\times 10^{3}$	$5.911\times 10^{3}$
	Std	$2.089\times 10^{3}$	$1.349\times 10^{3}$	$1.084\times 10^{3}$	$2.777\times 10^{3}$	$7.584\times 10^{2}$	$2.916\times 10^{3}$	$1.249\times 10^{3}$	$7.829\times 10^{2}$
	Rank	1	5	8	2	6	7	3	4
F23	Mean	$5.481\times 10^{2}$	$5.745\times 10^{2}$	$1.058\times 10^{3}$	$6.864\times 10^{2}$	$7.155\times 10^{2}$	$7.167\times 10^{2}$	$7.746\times 10^{2}$	$6.768\times 10^{2}$
	Std	$3.391\times 10^{1}$	$2.958\times 10^{1}$	$1.032\times 10^{2}$	$6.295\times 10^{1}$	$6.497\times 10^{1}$	$1.602\times 10^{1}$	$8.286\times 10^{1}$	$4.285\times 10^{1}$
	Rank	1	2	8	4	5	6	7	3
F24	Mean	$6.077\times 10^{2}$	$6.158\times 10^{2}$	$1.107\times 10^{3}$	$7.619\times 10^{2}$	$9.151\times 10^{2}$	$8.544\times 10^{2}$	$8.897\times 10^{2}$	$8.415\times 10^{2}$
	Std	$2.150\times 10^{1}$	$2.862\times 10^{1}$	$1.593\times 10^{2}$	$6.102\times 10^{1}$	$1.127\times 10^{2}$	$1.639\times 10^{1}$	$1.051\times 10^{2}$	$7.836\times 10^{1}$
	Rank	1	2	8	3	7	5	6	4
F25	Mean	$5.389\times 10^{2}$	$5.919\times 10^{2}$	$5.528\times 10^{2}$	$5.445\times 10^{2}$	$5.441\times 10^{2}$	$6.305\times 10^{2}$	$6.296\times 10^{2}$	$6.066\times 10^{2}$
	Std	$3.518\times 10^{1}$	$4.090\times 10^{1}$	$6.648\times 10^{1}$	$5.298\times 10^{1}$	$4.088\times 10^{1}$	$9.853\times 10^{0}$	$9.753\times 10^{1}$	$1.093\times 10^{2}$
	Rank	1	5	4	3	2	8	7	6
F26	Mean	$2.154\times 10^{3}$	$2.178\times 10^{3}$	$6.625\times 10^{3}$	$2.651\times 10^{3}$	$3.437\times 10^{3}$	$4.027\times 10^{3}$	$3.908\times 10^{3}$	$3.887\times 10^{3}$
	Std	$5.720\times 10^{2}$	$1.091\times 10^{3}$	$1.550\times 10^{3}$	$2.472\times 10^{3}$	$1.216\times 10^{3}$	$1.575\times 10^{2}$	$7.393\times 10^{2}$	$5.281\times 10^{2}$
	Rank	1	2	8	3	4	7	6	5
F27	Mean	$5.908\times 10^{2}$	$7.179\times 10^{2}$	$1.110\times 10^{3}$	$8.013\times 10^{2}$	$7.182\times 10^{2}$	$7.565\times 10^{2}$	$8.801\times 10^{2}$	$7.704\times 10^{2}$
	Std	$4.175\times 10^{1}$	$5.570\times 10^{1}$	$1.908\times 10^{2}$	$9.243\times 10^{1}$	$1.011\times 10^{2}$	$2.393\times 10^{1}$	$1.375\times 10^{2}$	$7.289\times 10^{1}$
	Rank	1	2	8	6	3	4	7	5
F28	Mean	$4.931\times 10^{2}$	$5.293\times 10^{2}$	$5.492\times 10^{2}$	$4.919\times 10^{2}$	$4.951\times 10^{2}$	$9.435\times 10^{2}$	$1.037\times 10^{3}$	$1.603\times 10^{3}$
	Std	$2.780\times 10^{1}$	$3.658\times 10^{1}$	$1.708\times 10^{2}$	$2.869\times 10^{1}$	$2.460\times 10^{1}$	$9.004\times 10^{1}$	$5.562\times 10^{2}$	$9.338\times 10^{2}$
	Rank	2	4	5	1	3	6	7	8
F29	Mean	$9.710\times 10^{2}$	$9.082\times 10^{2}$	$2.625\times 10^{3}$	$1.829\times 10^{3}$	$1.321\times 10^{3}$	$1.148\times 10^{3}$	$1.140\times 10^{3}$	$1.752\times 10^{3}$
	Std	$2.522\times 10^{2}$	$2.461\times 10^{2}$	$4.985\times 10^{2}$	$3.785\times 10^{2}$	$3.871\times 10^{2}$	$1.323\times 10^{2}$	$4.519\times 10^{2}$	$4.651\times 10^{2}$
	Rank	2	1	8	7	5	4	3	6
F30	Mean	$8.843\times 10^{5}$	$2.723\times 10^{6}$	$4.030\times 10^{6}$	$1.007\times 10^{7}$	$9.957\times 10^{5}$	$2.838\times 10^{6}$	$7.757\times 10^{6}$	$6.340\times 10^{6}$
	Std	$1.409\times 10^{5}$	$7.141\times 10^{5}$	$2.055\times 10^{6}$	$2.037\times 10^{6}$	$2.174\times 10^{5}$	$5.180\times 10^{5}$	$9.865\times 10^{6}$	$9.796\times 10^{6}$
	Rank	1	3	5	8	2	4	7	6

**Table 6:** Overall ranks on the 29 CEC 2017 functions at D=30 and D=50.

**Algorithm**	D=30			D=50		
	**Average Rank**	**First Place**	**Top-3**	**Average Rank**	**First Place**	**Top-3**
GRACE-PSO	1.2414	23	29	1.3793	21	28
AWPSO	3.5517	1	17	3.4828	1	16
APSO	4.3103	0	7	4.0690	2	9
VPPSO	4.8966	0	9	4.5517	2	11
AFMPSO	5.1724	5	10	5.5517	1	6
PSO	5.2069	0	8	5.4483	0	10
CPSO	5.5862	0	3	5.5862	1	4
CLPSO	6.0345	0	4	5.9310	1	3

**Table 7:** Average rank by CEC 2017 function category at D=30.

Algorithm	Unimodal	Multimodal	Hybrid	Composition
GRACE-PSO	1.000	1.143	1.600	1.000
AWPSO	4.000	3.714	3.300	3.600
AFMPSO	3.000	5.857	3.900	6.400
VPPSO	4.000	6.143	5.100	4.000
APSO	3.500	3.714	4.500	4.700
CLPSO	7.500	6.857	5.400	5.800
PSO	6.500	3.857	6.000	5.100
CPSO	6.500	4.714	6.200	5.400

**Table 8:** Omnibus rank tests across the 29 functions.

*D*	Friedman $\chi^{2}$	df	*p*	Iman–Davenport *F*	df	*p*	Nemenyi CD
30	78.2874	7	$3.08\times 10^{-14}$	17.5768	7, 196	$5.29\times 10^{-18}$	1.9497
50	78.2759	7	$3.09\times 10^{-14}$	17.5726	7, 196	$5.33\times 10^{-18}$	1.9497

**Table 9:** Pairwise comparisons with GRACE-PSO across the 29 CEC 2017 functions. Positive $r_{\mathrm{rb}}$ favors GRACE-PSO.

*D*	Competitor	Average Rank	Mean W/T/L	Holm *p*	$r_{\mathrm{rb}}$	Function-Level Holm W/T/L
30	AWPSO	3.5517	28/0/1	$5.99\times 10^{-6}$	0.899	18/11/0
30	AFMPSO	5.1724	24/0/5	0.00922	0.545	22/4/3
30	VPPSO	4.8966	28/0/1	$5.99\times 10^{-6}$	0.890	20/8/1
30	APSO	4.3103	29/0/0	$2.61\times 10^{-8}$	1.000	22/7/0
30	CLPSO	6.0345	29/0/0	$2.61\times 10^{-8}$	1.000	26/3/0
30	PSO	5.2069	29/0/0	$2.61\times 10^{-8}$	1.000	23/6/0
30	CPSO	5.5862	29/0/0	$2.61\times 10^{-8}$	1.000	28/1/0
50	AWPSO	3.4828	27/0/2	$1.30\times 10^{-5}$	0.867	20/9/0
50	AFMPSO	5.5517	27/0/2	$1.30\times 10^{-5}$	0.867	20/8/1
50	VPPSO	4.5517	27/0/2	$4.15\times 10^{-6}$	0.908	19/9/1
50	APSO	4.0690	27/0/2	$1.30\times 10^{-7}$	0.982	17/12/0
50	CLPSO	5.9310	27/0/2	$3.77\times 10^{-6}$	0.917	26/3/0
50	PSO	5.4483	29/0/0	$2.61\times 10^{-8}$	1.000	24/5/0
50	CPSO	5.5862	28/0/1	$1.12\times 10^{-7}$	0.986	26/3/0

**Table 10:** AUC of intergroup centroid distance at D=30. Values are medians [Q1, Q3] over ten runs; positive $r_{\mathrm{rb}}$ favors GRACE-PSO.

Interval	Function	GRACE-PSO	PSO	$r_{\mathrm{rb}}$/Holm *p*	APSO	$r_{\mathrm{rb}}$/Holm *p*
Full	F1	0.00959 [0.00935, 0.01008]	0.00852 [0.00835, 0.00872]	0.96/$9.90\times 10^{-4}$	0.00130 [0.00129, 0.00134]	1.00/$7.31\times 10^{-4}$
	F9	0.01226 [0.01212, 0.01259]	0.01162 [0.01144, 0.01181]	0.64/0.0345	0.00271 [0.00225, 0.00320]	1.00/$7.31\times 10^{-4}$
	F14	0.03151 [0.03041, 0.03235]	0.02069 [0.01951, 0.02166]	1.00/$7.31\times 10^{-4}$	0.02104 [0.01906, 0.02504]	1.00/$7.31\times 10^{-4}$
	F22	0.01176 [0.01155, 0.01199]	0.00872 [0.00837, 0.01731]	0.36/0.1859	0.00141 [0.00136, 0.00153]	1.00/$7.31\times 10^{-4}$
Final 25%	F1	0.00370 [0.00322, 0.00484]	$1.96\times 10^{-5}$ [$9.84\times 10^{-6}$, $2.85\times 10^{-5}$]	1.00/$7.31\times 10^{-4}$	$2.42\times 10^{-4}$ [$2.07\times 10^{-4}$, $2.84\times 10^{-4}$]	1.00/$7.31\times 10^{-4}$
	F9	0.00886 [0.00877, 0.00892]	0.00268 [0.00231, 0.00320]	0.98/$7.38\times 10^{-4}$	$3.01\times 10^{-4}$ [$2.34\times 10^{-4}$, $4.77\times 10^{-4}$]	1.00/$7.31\times 10^{-4}$
	F14	0.01448 [0.01256, 0.01569]	0.01829 [0.01739, 0.01995]	−0.48/0.0757	0.01871 [0.01525, 0.02531]	−0.48/0.0757
	F22	0.00917 [0.00912, 0.00943]	$5.90\times 10^{-4}$ [$1.57\times 10^{-4}$, 0.00839]	0.78/0.0072	$2.05\times 10^{-4}$ [$1.77\times 10^{-4}$, $2.33\times 10^{-4}$]	1.00/$7.31\times 10^{-4}$

**Table 11:** Ablation results for the six configurations. W/T/L and rank-biserial correlation compare GRACE-PSO with each configuration across the 29 functions; reported *p*-values are Holm adjusted.

Configuration	Average Rank	W/T/L	Holm *p*	$r_{\mathrm{rb}}$
GRACE-PSO	2.345	–	–	–
GL+SR (w/o CA)	2.966	18/0/11	0.325	0.209
GroupOnly	3.034	19/0/10	0.05003	0.531
GL+CA (w/o SR)	3.172	20/0/9	0.255	0.324
NonRank	3.586	20/0/9	0.193	0.393
PSO	5.897	29/0/0	$1.28\times 10^{-5}$	1.000

**Table 12:** Function-wise mean final errors for different values of *K*.

Function	K=2	K=4	K=6	K=8	K=10
F1	$3.932\times 10^{3}$	$3.745\times 10^{3}$	$2.715\times 10^{3}$	$3.250\times 10^{3}$	$1.983\times 10^{3}$
F3	$4.177\times 10^{-4}$	$1.724\times 10^{-3}$	$1.800\times 10^{-3}$	$1.453\times 10^{-2}$	$1.472\times 10^{-2}$
F4	$7.691\times 10^{1}$	$7.464\times 10^{1}$	$7.012\times 10^{1}$	$7.333\times 10^{1}$	$7.195\times 10^{1}$
F5	$5.500\times 10^{1}$	$5.895\times 10^{1}$	$4.991\times 10^{1}$	$4.673\times 10^{1}$	$5.143\times 10^{1}$
F6	$8.573\times 10^{-2}$	$9.573\times 10^{-2}$	$2.353\times 10^{-2}$	$1.748\times 10^{-2}$	$1.801\times 10^{-2}$
F7	$9.699\times 10^{1}$	$9.222\times 10^{1}$	$8.700\times 10^{1}$	$8.450\times 10^{1}$	$9.170\times 10^{1}$
F8	$5.666\times 10^{1}$	$5.331\times 10^{1}$	$5.640\times 10^{1}$	$4.950\times 10^{1}$	$4.769\times 10^{1}$
F9	$6.042\times 10^{0}$	$2.829\times 10^{0}$	$2.689\times 10^{0}$	$1.968\times 10^{0}$	$2.702\times 10^{0}$
F10	$3.019\times 10^{3}$	$2.885\times 10^{3}$	$2.892\times 10^{3}$	$2.906\times 10^{3}$	$3.065\times 10^{3}$
F11	$9.383\times 10^{1}$	$7.744\times 10^{1}$	$7.220\times 10^{1}$	$6.938\times 10^{1}$	$6.873\times 10^{1}$
F12	$3.241\times 10^{4}$	$2.385\times 10^{4}$	$2.669\times 10^{4}$	$2.360\times 10^{4}$	$2.613\times 10^{4}$
F13	$1.275\times 10^{4}$	$1.093\times 10^{4}$	$1.218\times 10^{4}$	$1.359\times 10^{4}$	$1.583\times 10^{4}$
F14	$4.549\times 10^{3}$	$4.516\times 10^{3}$	$4.422\times 10^{3}$	$3.781\times 10^{3}$	$3.723\times 10^{3}$
F15	$5.187\times 10^{3}$	$4.363\times 10^{3}$	$4.169\times 10^{3}$	$5.989\times 10^{3}$	$5.195\times 10^{3}$
F16	$6.743\times 10^{2}$	$5.574\times 10^{2}$	$5.877\times 10^{2}$	$5.162\times 10^{2}$	$5.629\times 10^{2}$
F17	$1.773\times 10^{2}$	$1.832\times 10^{2}$	$1.499\times 10^{2}$	$1.293\times 10^{2}$	$1.664\times 10^{2}$
F18	$1.063\times 10^{5}$	$1.032\times 10^{5}$	$1.202\times 10^{5}$	$1.334\times 10^{5}$	$1.301\times 10^{5}$
F19	$7.012\times 10^{3}$	$6.441\times 10^{3}$	$5.829\times 10^{3}$	$5.838\times 10^{3}$	$5.107\times 10^{3}$
F20	$2.352\times 10^{2}$	$2.075\times 10^{2}$	$1.947\times 10^{2}$	$1.907\times 10^{2}$	$2.031\times 10^{2}$
F21	$2.567\times 10^{2}$	$2.515\times 10^{2}$	$2.534\times 10^{2}$	$2.473\times 10^{2}$	$2.529\times 10^{2}$
F22	$6.557\times 10^{2}$	$1.974\times 10^{2}$	$3.527\times 10^{2}$	$2.949\times 10^{2}$	$3.738\times 10^{2}$
F23	$4.078\times 10^{2}$	$4.044\times 10^{2}$	$4.104\times 10^{2}$	$4.035\times 10^{2}$	$4.032\times 10^{2}$
F24	$4.787\times 10^{2}$	$4.714\times 10^{2}$	$4.740\times 10^{2}$	$4.690\times 10^{2}$	$4.715\times 10^{2}$
F25	$3.928\times 10^{2}$	$3.914\times 10^{2}$	$3.876\times 10^{2}$	$3.874\times 10^{2}$	$3.905\times 10^{2}$
F26	$1.267\times 10^{3}$	$1.162\times 10^{3}$	$1.245\times 10^{3}$	$1.208\times 10^{3}$	$1.061\times 10^{3}$
F27	$5.171\times 10^{2}$	$5.130\times 10^{2}$	$5.119\times 10^{2}$	$5.133\times 10^{2}$	$5.113\times 10^{2}$
F28	$3.733\times 10^{2}$	$3.539\times 10^{2}$	$3.571\times 10^{2}$	$3.785\times 10^{2}$	$3.614\times 10^{2}$
F29	$6.344\times 10^{2}$	$5.719\times 10^{2}$	$5.694\times 10^{2}$	$5.569\times 10^{2}$	$5.693\times 10^{2}$
F30	$5.820\times 10^{3}$	$5.170\times 10^{3}$	$4.756\times 10^{3}$	$5.201\times 10^{3}$	$4.701\times 10^{3}$

**Table 13:** Function-wise mean final errors for different values of ρ.

Function	ρ=0.04	ρ=0.06	ρ=0.08	ρ=0.10	ρ=0.12
F1	$3.492\times 10^{3}$	$3.998\times 10^{3}$	$2.715\times 10^{3}$	$3.687\times 10^{3}$	$3.308\times 10^{3}$
F3	$8.540\times 10^{-4}$	$2.151\times 10^{-3}$	$1.800\times 10^{-3}$	$2.326\times 10^{-4}$	$1.013\times 10^{-3}$
F4	$7.132\times 10^{1}$	$7.151\times 10^{1}$	$7.012\times 10^{1}$	$7.788\times 10^{1}$	$7.336\times 10^{1}$
F5	$5.208\times 10^{1}$	$5.088\times 10^{1}$	$4.991\times 10^{1}$	$5.247\times 10^{1}$	$4.879\times 10^{1}$
F6	$3.616\times 10^{-2}$	$1.076\times 10^{-2}$	$2.353\times 10^{-2}$	$9.311\times 10^{-3}$	$5.623\times 10^{-3}$
F7	$9.357\times 10^{1}$	$8.992\times 10^{1}$	$8.700\times 10^{1}$	$9.183\times 10^{1}$	$9.166\times 10^{1}$
F8	$5.126\times 10^{1}$	$4.792\times 10^{1}$	$5.640\times 10^{1}$	$5.272\times 10^{1}$	$5.250\times 10^{1}$
F9	$3.354\times 10^{0}$	$5.951\times 10^{0}$	$2.689\times 10^{0}$	$2.509\times 10^{0}$	$1.647\times 10^{0}$
F10	$2.930\times 10^{3}$	$3.245\times 10^{3}$	$2.892\times 10^{3}$	$2.817\times 10^{3}$	$3.114\times 10^{3}$
F11	$6.849\times 10^{1}$	$6.935\times 10^{1}$	$7.220\times 10^{1}$	$8.621\times 10^{1}$	$7.689\times 10^{1}$
F12	$2.682\times 10^{4}$	$2.397\times 10^{4}$	$2.669\times 10^{4}$	$2.654\times 10^{4}$	$2.627\times 10^{4}$
F13	$1.257\times 10^{4}$	$1.299\times 10^{4}$	$1.218\times 10^{4}$	$1.211\times 10^{4}$	$1.191\times 10^{4}$
F14	$3.756\times 10^{3}$	$4.187\times 10^{3}$	$4.422\times 10^{3}$	$3.878\times 10^{3}$	$4.400\times 10^{3}$
F15	$4.463\times 10^{3}$	$4.118\times 10^{3}$	$4.169\times 10^{3}$	$4.021\times 10^{3}$	$3.939\times 10^{3}$
F16	$5.764\times 10^{2}$	$6.274\times 10^{2}$	$5.877\times 10^{2}$	$5.978\times 10^{2}$	$5.960\times 10^{2}$
F17	$1.649\times 10^{2}$	$1.586\times 10^{2}$	$1.499\times 10^{2}$	$1.620\times 10^{2}$	$1.711\times 10^{2}$
F18	$1.120\times 10^{5}$	$9.928\times 10^{4}$	$1.202\times 10^{5}$	$1.145\times 10^{5}$	$1.171\times 10^{5}$
F19	$5.888\times 10^{3}$	$5.750\times 10^{3}$	$5.829\times 10^{3}$	$5.725\times 10^{3}$	$5.723\times 10^{3}$
F20	$1.863\times 10^{2}$	$1.954\times 10^{2}$	$1.947\times 10^{2}$	$1.951\times 10^{2}$	$2.039\times 10^{2}$
F21	$2.538\times 10^{2}$	$2.572\times 10^{2}$	$2.534\times 10^{2}$	$2.533\times 10^{2}$	$2.547\times 10^{2}$
F22	$3.467\times 10^{2}$	$3.664\times 10^{2}$	$3.527\times 10^{2}$	$2.034\times 10^{2}$	$3.523\times 10^{2}$
F23	$4.080\times 10^{2}$	$4.036\times 10^{2}$	$4.104\times 10^{2}$	$4.047\times 10^{2}$	$4.067\times 10^{2}$
F24	$4.738\times 10^{2}$	$4.720\times 10^{2}$	$4.740\times 10^{2}$	$4.720\times 10^{2}$	$4.701\times 10^{2}$
F25	$3.878\times 10^{2}$	$3.878\times 10^{2}$	$3.876\times 10^{2}$	$3.892\times 10^{2}$	$3.898\times 10^{2}$
F26	$1.205\times 10^{3}$	$1.179\times 10^{3}$	$1.245\times 10^{3}$	$1.152\times 10^{3}$	$1.201\times 10^{3}$
F27	$5.123\times 10^{2}$	$5.138\times 10^{2}$	$5.119\times 10^{2}$	$5.134\times 10^{2}$	$5.114\times 10^{2}$
F28	$3.639\times 10^{2}$	$3.505\times 10^{2}$	$3.571\times 10^{2}$	$3.579\times 10^{2}$	$3.502\times 10^{2}$
F29	$5.611\times 10^{2}$	$5.568\times 10^{2}$	$5.694\times 10^{2}$	$5.671\times 10^{2}$	$5.889\times 10^{2}$
F30	$4.722\times 10^{3}$	$4.771\times 10^{3}$	$4.756\times 10^{3}$	$4.581\times 10^{3}$	$4.635\times 10^{3}$

**Table 14:** Function-wise mean final errors for different values of λ.

Function	λ=0.20	λ=0.30	λ=0.40	λ=0.60	λ=0.80
F1	$2.962\times 10^{3}$	$2.999\times 10^{3}$	$2.715\times 10^{3}$	$2.712\times 10^{3}$	$2.888\times 10^{3}$
F3	$3.967\times 10^{-3}$	$3.027\times 10^{-4}$	$1.800\times 10^{-3}$	$1.636\times 10^{-4}$	$1.756\times 10^{-4}$
F4	$7.248\times 10^{1}$	$7.266\times 10^{1}$	$7.012\times 10^{1}$	$6.632\times 10^{1}$	$7.113\times 10^{1}$
F5	$4.746\times 10^{1}$	$5.141\times 10^{1}$	$4.991\times 10^{1}$	$5.385\times 10^{1}$	$5.599\times 10^{1}$
F6	$7.806\times 10^{-3}$	$2.656\times 10^{-2}$	$2.353\times 10^{-2}$	$1.092\times 10^{-2}$	$9.002\times 10^{-3}$
F7	$9.372\times 10^{1}$	$9.091\times 10^{1}$	$8.700\times 10^{1}$	$9.049\times 10^{1}$	$9.647\times 10^{1}$
F8	$5.230\times 10^{1}$	$4.666\times 10^{1}$	$5.640\times 10^{1}$	$4.749\times 10^{1}$	$5.259\times 10^{1}$
F9	$3.769\times 10^{0}$	$2.707\times 10^{0}$	$2.689\times 10^{0}$	$2.911\times 10^{0}$	$2.349\times 10^{0}$
F10	$2.815\times 10^{3}$	$3.036\times 10^{3}$	$2.892\times 10^{3}$	$2.997\times 10^{3}$	$2.991\times 10^{3}$
F11	$6.895\times 10^{1}$	$7.577\times 10^{1}$	$7.220\times 10^{1}$	$7.920\times 10^{1}$	$7.133\times 10^{1}$
F12	$2.292\times 10^{4}$	$2.164\times 10^{4}$	$2.669\times 10^{4}$	$2.540\times 10^{4}$	$2.821\times 10^{4}$
F13	$1.310\times 10^{4}$	$1.223\times 10^{4}$	$1.218\times 10^{4}$	$1.244\times 10^{4}$	$1.208\times 10^{4}$
F14	$4.309\times 10^{3}$	$4.221\times 10^{3}$	$4.422\times 10^{3}$	$4.861\times 10^{3}$	$5.649\times 10^{3}$
F15	$4.523\times 10^{3}$	$4.181\times 10^{3}$	$4.169\times 10^{3}$	$4.501\times 10^{3}$	$3.941\times 10^{3}$
F16	$6.062\times 10^{2}$	$5.568\times 10^{2}$	$5.877\times 10^{2}$	$5.748\times 10^{2}$	$5.568\times 10^{2}$
F17	$1.566\times 10^{2}$	$1.601\times 10^{2}$	$1.499\times 10^{2}$	$1.570\times 10^{2}$	$1.611\times 10^{2}$
F18	$9.424\times 10^{4}$	$1.078\times 10^{5}$	$1.202\times 10^{5}$	$1.191\times 10^{5}$	$1.144\times 10^{5}$
F19	$5.587\times 10^{3}$	$5.781\times 10^{3}$	$5.829\times 10^{3}$	$5.696\times 10^{3}$	$5.853\times 10^{3}$
F20	$2.043\times 10^{2}$	$2.043\times 10^{2}$	$1.947\times 10^{2}$	$1.937\times 10^{2}$	$1.986\times 10^{2}$
F21	$2.492\times 10^{2}$	$2.530\times 10^{2}$	$2.534\times 10^{2}$	$2.504\times 10^{2}$	$2.517\times 10^{2}$
F22	$2.070\times 10^{2}$	$4.120\times 10^{2}$	$3.527\times 10^{2}$	$3.200\times 10^{2}$	$3.525\times 10^{2}$
F23	$4.042\times 10^{2}$	$4.028\times 10^{2}$	$4.104\times 10^{2}$	$4.082\times 10^{2}$	$4.066\times 10^{2}$
F24	$4.759\times 10^{2}$	$4.718\times 10^{2}$	$4.740\times 10^{2}$	$4.725\times 10^{2}$	$4.667\times 10^{2}$
F25	$3.878\times 10^{2}$	$3.880\times 10^{2}$	$3.876\times 10^{2}$	$3.878\times 10^{2}$	$3.877\times 10^{2}$
F26	$1.167\times 10^{3}$	$1.211\times 10^{3}$	$1.245\times 10^{3}$	$1.175\times 10^{3}$	$1.173\times 10^{3}$
F27	$5.114\times 10^{2}$	$5.134\times 10^{2}$	$5.119\times 10^{2}$	$5.122\times 10^{2}$	$5.128\times 10^{2}$
F28	$3.559\times 10^{2}$	$3.652\times 10^{2}$	$3.571\times 10^{2}$	$3.694\times 10^{2}$	$3.624\times 10^{2}$
F29	$5.619\times 10^{2}$	$5.692\times 10^{2}$	$5.694\times 10^{2}$	$5.577\times 10^{2}$	$5.721\times 10^{2}$
F30	$5.046\times 10^{3}$	$4.853\times 10^{3}$	$4.756\times 10^{3}$	$4.874\times 10^{3}$	$4.684\times 10^{3}$

**Table 15:** Settings of the two joint element-movement and phase-shift problems.

Problem	*N*	(L,P)	Dimension	Spacing Constraints
FIM-1	4	(3,3)	12	6
FIM-2	8	(6,6)	24	28

**Table 16:** Results on FIM-1 and FIM-2. Values are computed from the 30 channel-level medians; larger adjusted normalized power is better, whereas smaller average rank is better.

Algorithm	FIM-1			FIM-2		
	Mean ± Std	Median [Q1, Q3]	Rank	Mean ± Std	Median [Q1, Q3]	Rank
GRACE-PSO	0.7030 ± 0.0785	0.7054 [0.6691, 0.7506]	2.900	0.1842 ± 0.0396	0.1769 [0.1515, 0.2106]	2.433
AWPSO	0.6885 ± 0.0825	0.6663 [0.6289, 0.7488]	3.667	0.1537 ± 0.0331	0.1464 [0.1318, 0.1817]	4.933
AFMPSO	0.6159 ± 0.0950	0.6039 [0.5575, 0.6633]	5.800	0.1226 ± 0.0263	0.1167 [0.1006, 0.1499]	6.200
VPPSO	0.5428 ± 0.0727	0.5280 [0.4939, 0.5832]	7.000	0.1121 ± 0.0219	0.1061 [0.0935, 0.1313]	6.767
APSO	0.7068 ± 0.0774	0.7028 [0.6712, 0.7665]	2.533	0.1873 ± 0.0355	0.1762 [0.1596, 0.2102]	2.033
CLPSO	0.5011 ± 0.0666	0.4912 [0.4498, 0.5590]	7.967	0.0756 ± 0.0155	0.0718 [0.0628, 0.0885]	8.000
PSO	0.7067 ± 0.0730	0.6940 [0.6603, 0.7594]	2.633	0.1743 ± 0.0322	0.1687 [0.1489, 0.2034]	3.600
CPSO	0.6873 ± 0.0860	0.6772 [0.6240, 0.7426]	3.500	0.1880 ± 0.0332	0.1803 [0.1608, 0.2226]	2.033
Fixed-position PBF	0.1789 ± 0.1082	0.1649 [0.1067, 0.2159]	–	0.0403 ± 0.0258	0.0308 [0.0251, 0.0518]	–

**Table 17:** Paired comparisons of GRACE-PSO. Reported *p*-values are Holm-adjusted within each problem. W/T/L denotes the number of channel-level wins, ties, and losses of GRACE-PSO.

Problem	Competitor	*p*	$r_{\mathrm{rb}}$	W/T/L	Significant
FIM-1	AWPSO	0.3747	0.351	20/0/10	No
FIM-1	AFMPSO	$1.21\times 10^{-5}$	1.000	30/0/0	Yes
FIM-1	VPPSO	$1.21\times 10^{-5}$	1.000	30/0/0	Yes
FIM-1	APSO	0.9306	−0.123	13/0/17	No
FIM-1	CLPSO	$1.21\times 10^{-5}$	1.000	30/0/0	Yes
FIM-1	PSO	0.9306	−0.153	12/0/18	No
FIM-1	CPSO	0.3747	0.342	18/0/12	No
FIM-2	AWPSO	$1.21\times 10^{-5}$	1.000	30/0/0	Yes
FIM-2	AFMPSO	$1.21\times 10^{-5}$	1.000	30/0/0	Yes
FIM-2	VPPSO	$1.21\times 10^{-5}$	1.000	30/0/0	Yes
FIM-2	APSO	0.3057	−0.299	13/0/17	No
FIM-2	CLPSO	$1.21\times 10^{-5}$	1.000	30/0/0	Yes
FIM-2	PSO	0.0055	0.652	21/0/9	Yes
FIM-2	CPSO	0.3057	−0.217	13/0/17	No

## Data Availability

The source code of GRACE-PSO is provided as Supplementary Materials. Further inquiries can be directed to the corresponding author.

## Supplementary Materials

The following supporting information can be downloaded at https://www.mdpi.com/article/10.3390/biomimetics11090664/s1.

## Abbreviations

The following abbreviations are used in this manuscript:

AFMPSO	Adaptive Forgetting Multi-Strategy Particle Swarm Optimization
APSO	Adaptive Particle Swarm Optimization
AWPSO	Adaptive Weighted Particle Swarm Optimizer
CEC	Congress on Evolutionary Computation
CLPSO	Comprehensive Learning Particle Swarm Optimizer
CPSO	Cooperative Particle Swarm Optimization
CA	Coefficient Adaptation
ESE	Evolutionary State Estimation
FIM	Flexible Intelligent Metasurface
GRACE-PSO	Group Rank Assessment and Cooperative Evolution PSO
PSO	Particle Swarm Optimization
SR	Selective Reinitialization
VPPSO	Velocity Pausing Particle Swarm Optimization
